# Acetylation and accessibility of *Cryptococcus neoformans* cell wall chitosans influence the strength of host immune responses

**DOI:** 10.1016/j.tcsw.2026.100175

**Published:** 2026-05-20

**Authors:** Margareta J. Hellmann, Rajendra Upadhya, Evelyn Tchoub, Bruno M. Moerschbacher, Jennifer K. Lodge, Stefan Cord-Landwehr

**Affiliations:** a Institute for Biology and Biotechnology of Plants, University of Münster, 48143 Münster, Germany; b Department of Molecular Genetics and Microbiology, Duke University School of Medicine, Durham, NC, United States

**Keywords:** *Cryptococcus*, Chitosan, Chitin deacetylases, Cell wall, Host–pathogen interactions

## Abstract

The fungal pathogen *Cryptococcus neoformans* causes fatal cryptococcal meningitis, a significant global health risk. Unlike most fungi, *C. neoformans* expresses chitin deacetylases that convert its cell wall chitin into chitosan, helping to evade chitin-triggered host immunity. However, it is unclear how the properties and accessibility of these chitosans vary between strains and growth conditions, and how they influence the host immune response. To show how chitin deacetylase activity and growth conditions shape the properties of cell wall chitosans, we present the first comprehensive analysis of the chitosan content, fraction of acetylation (FA), enzymatic degradation products by host chitinases, and accessibility in various *Cryptococcus* strains, including chitin deacetylase mutants, cultivated under different conditions. Certain chitin deacetylase knockouts, as well as challenging growth conditions, lead to higher FAs and enhanced exposure, associated with stronger host immune responses. The response to cryptococcal chitin/chitosan ranges from low (resulting in cryptococcosis) to adequate (providing effective host defense) and excessive (leading to hyper-inflammation). Correlation between the strength of the immune response and the abundance of chitin-binding proteins was used to rapidly screen a multitude of strains and growth conditions for their immunogenic potential. Our approach provides insight into *Cryptococcus*-related disease caused by insufficient and excessive immune responses, and may accelerate the development of effective cell-based vaccines.

## Highlights

• First analysis of cryptococcal cell wall chitosans using state-of-the-art methods.
• Challenging conditions increase cell wall chitosans' acetylation and accessibility.
• Knock-out of chitin deacetylase(s) leads to higher acetylation and accessibility.
• Higher exposure and/or acetylation correlates with stronger host immunity.
• Results are relevant to develop cell-based vaccines against cryptococcosis.

## 1 Introduction

The basidiomycetous yeast *Cryptococcus neoformans* (*Cn*) was recently classified as the top-ranking “critical group” fungal pathogen by the World Health Organization (World Health Organization, 2022). The associated disease is a severe global health threat. Human contact with the fungus is frequent, but an insufficient host immune response following the inhalation of spores can result in acute infection of the respiratory tract (pulmonary cryptococcosis) with subsequent dissemination to the central nervous system (cryptococcal meningitis) (He et al., 2024; Levitz, 1991; Rodrigues et al., 1999; Tugume et al., 2023; Wiesner et al., 2015). Cryptococcal meningitis is most prevalent in patients with acquired immunodeficiency syndrome (AIDS), accounting for more than 150,000 annual cases, more than 110,000 of which are fatal, making the fungus responsible for 19% of AIDS-related deaths (Rajasingham et al., 2022). Cryptococcal disease is also becoming more prevalent in persons without HIV (Kiertiburanakul et al., 2006; Pyrgos et al., 2013), such as solid organ transplant recipients (Ramirez-Sanchez et al., 2022), and mortality remains high because it is difficult to treat fungal infections (Iyer et al., 2021; Patel et al., 2018). Most cases are attributed to the ubiquitous species *Cn* (formerly known as *Cn* var. *grubii*), but its close (formerly tropical or subtropical) relatives of the *Cryptococcus gattii* (*Cg*) species complex are gaining prominence because their endemic area is expanding, they cause more severe infection, and even affect immunocompetent individuals (Diniz-Lima et al., 2022; Hagen et al., 2015; Kidd et al., 2004). It should be mentioned that data from Chinese cryptococcosis patients suggest the ability to infect hosts with intact immunity also for *Cn* (Chen et al., 2008).

Vegetative fungal cells drive infection (Upadhya et al., 2018), and their success depends on the avoidance and/or dampening of the host immune response. This allows them to proliferate in the lung, survive within macrophages (Feldmesser et al., 2001; Feldmesser et al., 2000), enter the bloodstream, cross the blood–brain barrier, and invade the central nervous system (Lange et al., 2023; Rodrigues et al., 1999; Strickland and Shi, 2021). Effective defense against *Cn* mostly involves cell-mediated immunity (Feldmesser et al., 2001). The response is triggered when pathogen-associated molecular patterns (PAMPs) bind to pattern recognition receptors (PRRs) on innate immune cells, leading to phagocytosis and lysis, amplified by Th1 responses involving CD4^+^ and CD8^+^ T cells (Hill, 1992; Huffnagle et al., 1991; Kitai et al., 2021; Levitz and Specht, 2006; Sato and Kawakami, 2022). Molecules on the *Cn* cell surface therefore play a decisive role in host–pathogen interactions and immune evasion (Baker et al., 2007; Doering, 2009), particularly the extracellular components (Mukaremera, 2023; Wang et al., 2018). The outermost polysaccharide capsule, consisting of glucuronoxylomannan, glucuronoxylomannogalactan (formerly galactoxylomannan) and mannoproteins (Doering, 2009), is a key virulence factor (Casadevall et al., 2019; Cherniak and Sundstrom, 1994; Fromtling et al., 1982) that forms a single complex structure together with the fungal cell wall (Gilbert et al., 2010). The latter consists of glucans, chitin, chitosans and melanin, but the researchers still discuss about the precise layout (Diniz-Lima et al., 2022; Garcia-Rubio et al., 2020; He et al., 2024; Iyer et al., 2021; Sato and Kawakami, 2022). The role of melanin in virulence is well-established (Kwon-Chung et al., 1982; Liu et al., 2021; Pukkila-Worley et al., 2005), but there is growing evidence that cell wall chitosans also facilitate immune evasion.

Fungal cell wall chitin is a linear polymer of β-1,4-linked *N-*acetylglucosamine (GlcNAc) units, and its partial deacetylation yields chitosans consisting of both GlcNAc and deacetylated glucosamine (GlcN) units. This diverse family of biopolymers is characterized by three key parameters: chain length (degree of polymerization, DP), proportion of acetylated GlcNAc units (fraction of acetylation, FA), and distribution of these units along the chain (pattern of acetylation, PA) (Wattjes et al., 2020b). Cell wall chitosan is mostly restricted to the subkingdom Mucoromyceta (Lecointe et al., 2019), and in many fungal pathogens that infect plants the deacetylation of chitin to chitosan may confer a stealth mechanism to avoid enzymatic degradation and the PRR-based detection of chitin (Student et al., 2024). The same PAMP variation mechanism (Lange et al., 2023) can also be used by *Cryptococcus spp.*, similar to the masking of other PAMPs such as β-glucans (Garcia-Rubio et al., 2020). Despite the presence of genes for eight chitin synthases (Chs) and three Chs regulator proteins (Csr) in *Cn*, only the chitin chains synthesized by the Chs3-Csr2 complex are partially deacetylated to chitosan by chitin deacetylases (CDAs) (Banks et al., 2005).

The three GPI-anchored *Cn*CDAs (hereafter CDA1–3) are localized on the extracellular side of the plasma membrane and are thought to act directly on nascent chitin chains that emerge from the transmembrane Chs3 (Baker et al., 2007; Gilbert et al., 2012). A complete knockout of all three genes is necessary to obtain fully chitosan-deficient *Cn* mutants (*cda1Δ2Δ3Δ*). These are viable but exhibit impaired cell wall integrity, greater stress and temperature sensitivity, and a leaky melanin phenotype (Baker et al., 2007). Most importantly, all chitosan-deficient mutants (*cda1Δ2Δ3Δ*, *chs3Δ* and *csr2Δ*) still containing chitin are avirulent and rapidly cleared by the host (Baker et al., 2011). The combination of aberrant cell wall organization and missing deacetylation may expose chitin, allowing degradation by the three human chitinolytic enzymes chitotriosidase (CHIT1), acidic mammalian chitinase (AMCase) and human lysozyme (HL) (Hellmann et al., 2025), and/or recognition by PRRs such as Toll-like receptor 2 (TLR2) or Dectin-1 (Elieh Ali Komi et al., 2018; Esher et al., 2018; Gorzelanny et al., 2010; Upadhya et al., 2016). In *Cn*, CDA1 is the primary CDA responsible for virulence and is upregulated during infection. But unlike *cda1Δ2Δ3Δ* mutants, *cda1Δ* mutants still produce some chitosan and are not effectively cleared (Upadhya et al., 2018; Yu et al., 2021). CDA2, but not CDA3, can partially compensate for the loss of CDA1, and the double mutant *cda1Δ2Δ* can be cleared by the host whereas the double mutant *cda1Δ3Δ* behaves like *cda1Δ* (Upadhya et al., 2021). *Cg* R265 (VGII) has orthologs of CDA1–3, and *Cg*CDA1 is the most important for growth in complex media but *Cg*CDA3 is necessary for virulence (Lam et al., 2019).

In contrast to the GPI-anchored CDAs, the mutation of *Cn*CDA4 generates no discernible phenotype (Baker et al., 2007). This enzyme is secreted and may act in the extracellular space, and is the only *Cn*CDA whose mode of action has been analyzed in detail thus far (Hembach et al., 2020; Hembach et al., 2017). Surprisingly, CDA4 has an unconventional preference for GlcN units at its −1 subsite, meaning it preferentially acts on substrates that have already undergone partial deacetylation by other CDAs. Further deacetylation of chitosan oligomers by CDA4 diminishes their ability to function as PAMPs. This suggests that CDA4 may disarm chitosan oligomers released from the fungal cell wall by human chitinases, which could otherwise alert the host immune system (Hembach et al., 2020).

The efficacy of chitosan-deficient mutants as vaccines against *Cryptococcus* has been demonstrated in mice. Live or heat-killed *cda1Δ2Δ3Δ* cells provide protective immunity against a subsequent lethal challenge with wild-type (WT) *Cn* but are less effective against the WT *Cg* species complex (Lam et al., 2019; Upadhya et al., 2016). *Cn* + *Cg* coinfection models have shown that *Cg* R265 (VGII) does not suppress, but rather evades, the vaccine-generated immunity, which is mediated by a Th1 response (Hester et al., 2024). However, differentiating between cryptococcal infection or cryptococcosis (ineffective host immune response) on one hand and successful host defense (effective host immune response) on the other is an oversimplification. A third outcome, described as immune reconstitution inflammatory syndrome (IRIS), also results in disease, with mortality rates similar to those observed in patients with untreated cryptococcal meningitis (Ost et al., 2017). Typically, IRIS occurs during restoration of the immune system in previously immunosuppressed patients, leading to an excessive response that is damaging instead of protective (Perfect, 2012). Similarly, mice challenged with fungal mutants exposing PAMPs such as chitin may not always develop protective immunity, but can rapidly succumb to hyper-inflammation mediated by neutrophils (Hole et al., 2020; O'Meara et al., 2013; Ost et al., 2017; Upadhya et al., 2023). The challenge of an optimal balance between effective and excessive responses is particularly evident when comparing different chitosan-deficient *Cn* mutants: in mice, inoculation with *cda1Δ2Δ3Δ* cells (chitin derived from all Chs – including Chs3 – present) confers protective immunity whereas the same dose of living or heat-killed *chs3Δ* cells (only Chs3-derived chitin absent) results in host death within 36 h (Hole et al., 2020). Effective vaccination may favor a protective Th1 response as opposed to a disease-exacerbating Th2 response (Upadhya et al., 2016; Wiesner et al., 2015). The spectrum of responses, ranging from none (infection) to effective (successful defense) and excessive (hyper-inflammation) is a phenomenon observed in numerous infectious diseases. The corresponding general concept is known as the damage-response framework of microbial pathogenesis (Casadevall and Pirofski, 2003).

In this study, we characterized cryptococcal chitosans and their exposure using novel analytical techniques to investigate their role in evading an immune response in the human host. Building on evidence that growth conditions and the presence of chitosans determine the strength of PRR-mediated reactions in reporter cells, we conducted the first detailed, comparative analysis of WT *Cn* and all 15 possible CDA knockout mutants cultivated under diverse conditions, focusing on the FA and mass fraction. Due to its increasing relevance for global health and its elevated pathogenicity (Diniz-Lima et al., 2022; Kidd et al., 2004), we also included WT *Cg* R265 (VGII) into our comparison. Imaging a fluorescent chitin-binding protein (Fuenzalida et al., 2014) enabled us to establish a model for the correlation between the accessibility and FA of chitin/chitosan in the *Cn* cell wall and the corresponding host immune response reported in previous work. This supports the proposal that chitin/chitosan exposure and FA are important for immune detection and also demonstrates that imaging can be used to anticipate the immune response against specific fungal cells, thereby facilitating larger screens prior to animal experiments. Finally, we investigated how different *Cn* strains grow in the presence of chitinolytic enzymes, and which products are released from *Cn* cell walls by the human chitinases CHIT1 and AMCase, adding the facet of fungal chitosan degradation by host enzymes. A greater understanding of chitosan as a *Cryptococcus* virulence factor provides more insight into pathogen–host interactions and may facilitate the development of more effective treatments and cell-based vaccines.

## 2 Results and discussion

### 2.1 PRR-mediated responses are influenced by changes in chitosans or growth conditions

**Fig. 1:**
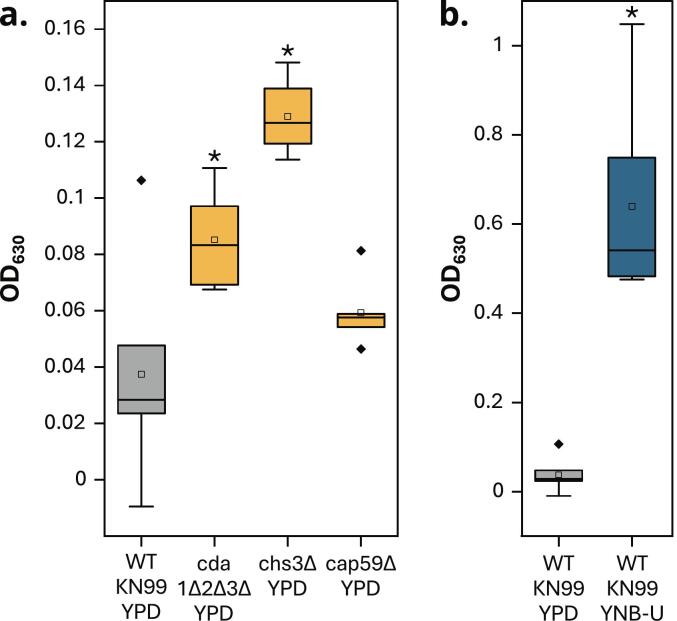
**Responses of NF-κB-SEAP macrophage reporter cells expressing various PRRs to different *Cn* cells.** The SEAP reporter gene is induced by NF-κB activation, and SEAP activity is quantified by spectrophotometry at 630 nm using Quanti-Blue as the substrate. WT *Cn* cultivated in YPD was compared to (**a**) mutant strains grown under the same conditions or (**b**) the same WT strain grown in YNB-U. Each strain and condition was tested in two independent experiments (*N* = 2) with three replicates each (*n* = 3). The whiskers show the range of data points within 1.5× the interquartile range. Statistical significance (*p < 0.01) was determined by one-way ANOVA with Tukey's multiple comparisons test between each sample and WT KN99 YPD. (For interpretation of the references to color in this figure legend, the reader is referred to the web version of this article.)

As a starting point, we aimed to provide proof of principle that the inability to convert chitin to chitosans, the absence of a capsule, as well as the conditions of fungal growth have an effect on the response of murine PRRs to cryptococcal cells. We assessed four fungal strains grown under two different conditions (complete YPD medium or minimal YNB-U medium) for their ability to elicit host immune responses. To this end, we used an NF-κB secreted embryonic alkaline phosphatase (SEAP) macrophage reporter cell line expressing PRRs such as Dectin-1 and most TLRs, including the proposed mammalian chitin receptor TLR2 (Elieh Ali Komi et al., 2018; Fuchs et al., 2018; Gorzelanny et al., 2010). The chitosan-deficient, but chitin-containing, strains *cda1Δ2Δ3Δ* and *chs3Δ* grown in YPD medium induced significantly stronger reactions than WT *Cn* and the capsule-deficient mutant *cap59Δ* (Fig. 1a). This supports the hypothesis that chitin-to-chitosan conversion contributes to the fungal stealth mechanism that avoids host immunity and promotes successful infections (Baker et al., 2011; Upadhya et al., 2016). In contrast, yeast cells lacking this conversion can trigger host immune responses that range from protective (*e.g.*, conferred by strain *cda1Δ2Δ3Δ* in YPD) to hyper-inflammatory (*e.g.*, conferred by strain *chs3Δ* in YPD), the latter masking a simultaneous protective response (Hole et al., 2020; Upadhya et al., 2016). In our experimental setting, the absence of chitin-to-chitosan conversion activated the NF-κB pathway in the reporter cell lines much more strongly than the absence of the capsule. Although WT *Cn* showed low immunoactivity when cultivated in YPD medium, it induced very strong PRR signaling when cultivated in YNB-U (Fig. 1b). This agrees with previous work showing that WT *Cn* grown in YNB-U, resulting in altered cell wall organization and decreased cell wall chitosan content, causes hyper-inflammatory responses when used to vaccinate mice (Upadhya et al., 2023).

### 2.2 *Cn* growth is impaired under host-mimicking conditions, especially in the absence of chitosan

**Fig. 2:**
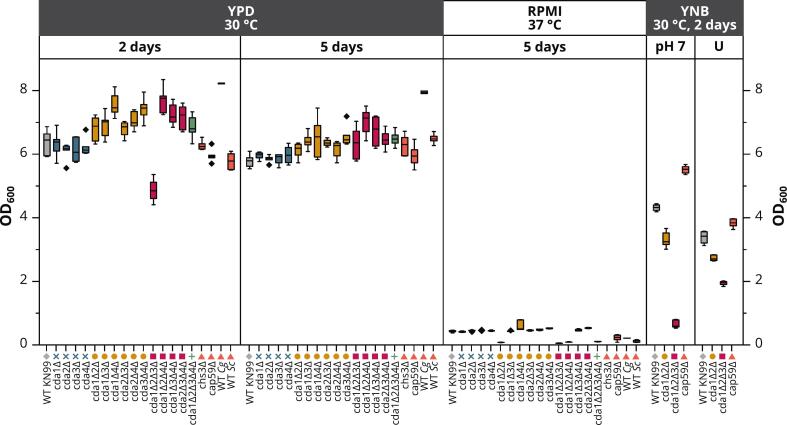
**Growth of *Cryptococcus* strains under different conditions.** For each *Cn* strain and condition, biological triplicates were cultivated (*N* = 3) and the OD_600_ was measured in technical duplicates for each (*n* = 6). For WT *Cg* R265 (VGII), only one biological replicate was cultivated for each condition and was measured as a technical duplicate (*n* = 2). The whiskers show the range of data points within 1.5× the interquartile range. The RPMI panel is shown in more detail in Fig. S1.

After establishing an initial link between receptor signaling and cell wall composition and architecture, we next sought to examine in greater detail the relationships among fungal chitosans, cultivation conditions, and host immune responses. Prior studies have shown that although the three CDAs exhibit functional redundancy when fungal cells are grown in YPD, the activity of individual CDAs becomes essential under nutrient-limiting or host-mimicking conditions (Upadhya et al., 2021). Accordingly, we aimed to define the specific contribution of each CDA to chitosan production in different growth media, and started by comparing the influence of cultivation conditions and CDA knockouts on fungal growth. We cultivated WT *Cn* KN99 (KN99α) and all 15 possible CDA knockout mutants, as well as the chitosan-deficient *Cn* mutant *chs3Δ*, the acapsular *Cn* mutant *cap59Δ*, WT *Cg* R265 (VGII), and the model yeast *Saccharomyces cerevisiae* (*Sc*). We first compared the growth of all 20 strains by collecting samples after 2 and 5 days of growth at 30 °C in complete medium (YPD), or 5 days of growth under host-mimicking conditions at 37 °C in cell culture medium (RPMI). Four *Cn* strains – WT KN99, *cda1Δ2Δ*, *cda1Δ2Δ3Δ* and *cap59Δ* – were also cultivated in minimal yeast medium (YNB) at 30 °C, either buffered to pH 7 (YNB-pH 7) or left unbuffered (YNB-U). Nearly all strains reached OD_600_ values of 6–8 after 2 days in YPD medium and no further increase was observed after 5 days (Fig. 2). The triple mutant *cda1Δ2Δ3Δ* grew more slowly, but also reached a comparable OD_600_ after 5 days, whereas the quadruple mutant *cda1Δ2Δ3Δ4Δ* behaved like the other strains. WT *Cg* R265 (VGII) achieved the highest OD_600_ (> 8) already after 2 days. Compared to YPD, the growth of all strains was much slower under host-mimicking conditions, especially mutants lacking both *CDA1* and *CDA2* (*cda1Δ2Δ*, *cda1Δ2Δ3Δ*, *cda1Δ2Δ4Δ* and *cda1Δ2Δ3Δ4Δ*) (Fig. S1). At these conditions, *chs3Δ* did not grow at all which is consistent with its reported temperature-sensitive phenotype at 37 °C (Hole et al., 2020). In YNB-pH 7, the final OD_600_ varied widely between strains, ranging from <1 for *cda1Δ2Δ3Δ* to 5–6 for *cap59Δ*. A similar trend was observed in YNB-U but with a narrower range of OD_600_ = 2–4.

The absence of cell wall chitosan was previously shown to limit growth and confer stress sensitivity, potentially due to reduced cell wall plasticity or melanin-binding ability (Baker et al., 2007; Banks et al., 2005). We observed that this effect was minimal at the optimal temperature (30 °C) and in complete medium (YPD), but mutants missing several CDAs (described as higher-order mutants hereafter) showed growth restriction at elevated temperature (37 °C), altered pH and/or in less nutrient-rich media. To gain further insight into the effects of these three factors, we screened the strains using various combinations of temperature and medium (Fig. S2). The most robust strains (WT and *cda1Δ3Δ*) showed slightly impaired growth at 37 °C compared to 30 °C in YPD and YNB-pH 7, confirming that temperature stress contributes to the poor growth performance of all strains under host-mimicking conditions. However, suboptimal pH was even more significant. A pH < 8.5 in RPMI medium (achieved by adding 5% CO_2_ to the air) was necessary for the growth of all *Cn* strains, whereas strains growing in YPD at pH 4.5–5.5 exhibited particularly robust growth. In comparison to WT *Cn*, the higher-order mutants were much more sensitive to both temperature and pH stress, explaining their limited growth in RPMI at high temperature and pH. The final low pH of YNB-U (∼2) or the high pH of YNB-pH 7 (∼7) may also cause pH stress, leading to the impaired growth of strain *cda1Δ2Δ3Δ* under both conditions (Fig. 2). Moreover, aeration and/or agitation increased growth, explaining the greater differential in the growth of WT *Cn* between YPD and RPMI in Fig. 2 (cultured at 300 rpm and 0 rpm, respectively) than Fig. S2 (both cultured at 0 rpm).

### 2.3 Chitin/chitosan content and FA vary widely between mutants and growth conditions

**Fig. 3:**
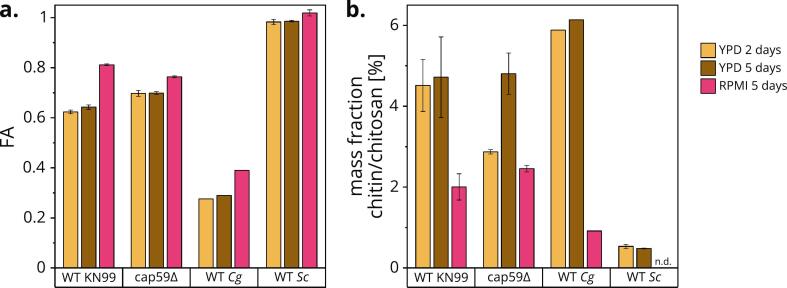
**FA (a) and content of chitin and chitosan (b) of strains without CDA knockouts grown under different conditions.** Each strain and condition was analyzed as biological triplicates (*N* = 3) except WT *Cg* R265 (VGII) (*N* = 1). Due to the very small amount of material obtained from *Sc* under RPMI conditions, no mass fraction was determined (n.d.). Data are means ± standard deviations.

We next aimed to characterize the chitin and chitosans of these mutants under distinct environmental conditions by analyzing purified dry cell wall material by highly sensitive and specific enzymatic MS fingerprinting. The cell walls of strains cultivated under different growth conditions varied in terms of FA and content of chitin and/or chitosan, as measured by the mass fraction determined by quantitative MS (Fig. S3). We want to emphasize that the mass fraction of chitin and/or chitosan (chitin/chitosan) quantifies all chitinous molecules of FA 0–1, including polyglucosamine, chitosan, and chitin. The strains without CDA knockouts (WT *Cn*, *cap59Δ*, WT *Cg* R265 (VGII) and WT *Sc*) showed extensive differences in YPD (Fig. 3), but overall, cultivation in RPMI (37 °C, 0 rpm) increased the cell wall FA and strongly reduced the cell wall chitin/chitosan content compared to YPD (30 °C, 300 rpm). In YPD, only *cap59Δ* accumulated more chitin/chitosan between day 2 and day 5 of cultivation. Consistent with previous data, *Sc* possessed a fully acetylated chitin cell wall (FA ≈ 1) and a very low mass fraction of chitinous material (Banks et al., 2005) (Table S1). In contrast, WT *Cn* under YPD conditions was characterized by an FA of 0.62–0.64 and a chitin/chitosan content of 4.5–4.7%, higher than the FAs of 0.22–0.47 and chitin/chitosan content of 1.6–3.4% reported previously under the same or similar conditions (Baker et al., 2007; Banks et al., 2005; Lam et al., 2019; Upadhya et al., 2023). Another study reported a chitin/chitosan mass fraction of 9.8% (He et al., 2024). The FA and chitin/chitosan content of the *cap59Δ* mutant was similar for each growth condition, whereas the cell wall of WT *Cg* R265 (VGII) was much more extensively deacetylated. These data indicate that the FA and chitin/chitosan content of WT *Cg* R265 (VGII) under YPD conditions remained near constant between day 2 and day 5 of cultivation (FA 0.28–0.29, mass fraction 5.9–6.2%), contrasting with the changing values reported elsewhere for the same cultivation period (FA 0.11–0.33, mass fraction 2.4–6.1) (Lam et al., 2019). The lower FA of *Cg* R265 (VGII) cell walls may contribute to its superior immune evasion after vaccination (Hester et al., 2024), and may play a role in the greater pathogenicity of the *Cg* species complex compared to *Cn* (Diniz-Lima et al., 2022).

**Fig. 4:**
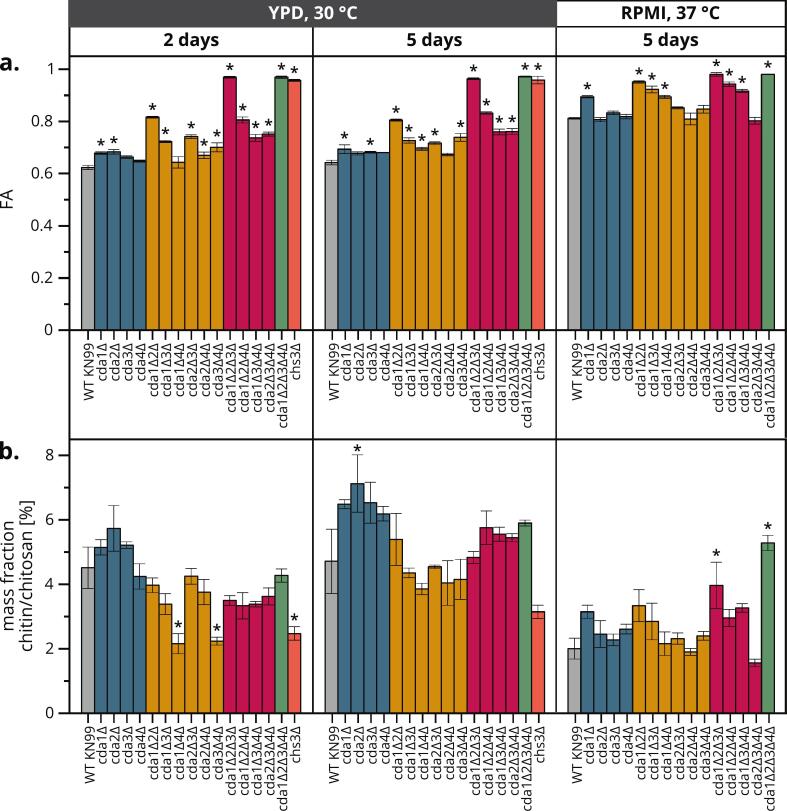
**FA (a) and content of chitin and chitosan (b) of *Cn* strains with chitosan-related knockouts grown under different conditions.** Single, double, triple and quadruple CDA mutants are shown in blue, yellow, red and green, respectively. For each strain and condition, we analyzed biological triplicates (N = 3). Data are means ± standard deviations. Statistical significance (*p < 0.01) was determined by one-way ANOVA with Tukey's multiple comparisons test between mutants and WT KN99 for each cultivation condition. (For interpretation of the references to color in this figure legend, the reader is referred to the web version of this article.)

To determine the contribution of each individual CDA on the overall chitosan content under different growth conditions, we characterized the cell wall chitosan of all 15 CDA knockout combinations and *chs3Δ* under the same set of growth conditions (Fig. 4). Again, RPMI conditions led to overall higher FAs and lower chitin/chitosan levels compared to YPD conditions over 5 days. Considering the stealth hypothesis, where the deacetylation of cell wall chitin helps to avoid a host immune response, it was surprising that the host-mimicking conditions increased the cell wall FA. Highly but not fully acetylated cell walls may allow cells to survive the more stressful and demanding temperature, pH and medium conditions during RPMI cultivation. This constitutes another example of cell wall plasticity that helps fungi to adapt to changing environments (Garcia-Rubio et al., 2020). Alternatively, the activity of the three membrane-associated CDAs 1–3 could be impaired under these conditions. There was no substantial change in FA when the incubation of strains in YPD was extended from 2 to 5 days, but the chitin/chitosan content increased during this interval. The accumulation of more cell wall components in aging yeast cells has been reported previously (Silva et al., 2022). Earlier time points of YPD cultivation may need to be tested to observe this effect in WT *Cn*.

When comparing the mutants grown in YPD, the FA exceeded 0.8 only in mutants lacking both CDA1 and CDA2 (*cda1Δ2Δ*, *cda1Δ2Δ3Δ*, *cda1Δ2Δ4Δ* and *cda1Δ2Δ3Δ4Δ*) and in *chs3Δ*. Under host-mimicking conditions, these specific mutants showed severely impaired growth (Fig. 2) and exceptionally high cell wall FA values (Fig. 4, right). Notably, all mutants showing significantly higher FA values than WT *Cn* in RPMI are lacking CDA1. These results are consistent with the observation that CDA1 is the critical and strongest upregulated CDA in *Cn* during infection (Upadhya et al., 2018) and CDA2 compensates for its loss more than CDA3 (Upadhya et al., 2021). As in previous reports (Baker et al., 2007), *CDA4* knockout did not result in a distinct phenotype. The only FA values available in the literature were determined under YPD conditions (2 days) for five of the mutants, and they also show FAs close to 1 for *cda1Δ2Δ3Δ*, *cda1Δ2Δ3Δ4Δ* and *chs3Δ* but lower values than our study for *cda1Δ2Δ* and *cda1Δ2Δ4Δ* (0.55 and 0.52, respectively) (Baker et al., 2007), similar to the trend described above for WT *Cn*. Nevertheless, this study and our data concur that cell wall FAs in these latter two mutants were similar to each other and considerably higher than the FA for WT *Cn*.

The comparison of chitin/chitosan content between mutants showed barely consistent trends, but under RPMI conditions only the chitin mass fraction of *cda1Δ2Δ3Δ* and *cda1Δ2Δ3Δ4Δ* differed significantly from WT *Cn*, as previously shown for these mutants grown in YPD for 70 h at 25 °C (Baker et al., 2007). Chitosan may therefore be part of a feedback loop controlling chitin synthesis. The activity of the chitin-synthesizing complexes may not be properly inhibited in chitosan-deficient mutants, causing chitin levels to increase. For comparison, only limited data are available for the FA and total chitin/chitosan content of *Cryptococcus* WT and CDA mutants (Baker et al., 2007; Banks et al., 2005; Lam et al., 2019; Upadhya et al., 2023), but more studies have determined the molar GlcN content (Baker et al., 2007; Banks et al., 2005; Gilbert et al., 2012; Hole et al., 2020; Lam et al., 2019; Upadhya et al., 2023; Upadhya et al., 2021; Upadhya et al., 2018) (Table S1). Although the absolute values are not always consistent between our data and these studies, the overall trends of high, medium or low GlcN content between mutants and cultivation conditions are very similar.

**Fig. 5:**
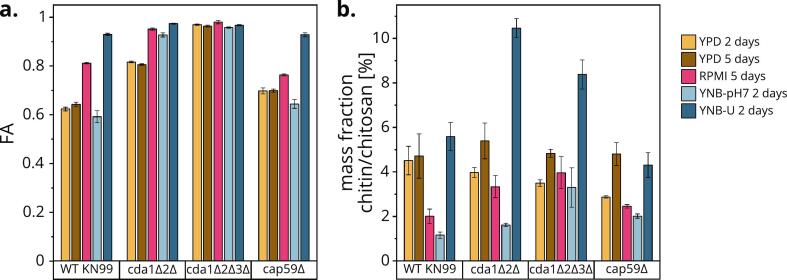
**FA (a) and content of chitin and chitosan (b) of selected strains grown in YNB.** For each strain and condition, we analyzed biological triplicates (*N* = 3). Data are means ± standard deviations.

A previous study reported a strong effect of the medium on the cell wall composition of WT *Cn* cells; especially cultivation in YNB-U resulted in altered cell wall architecture and decreased cell wall chitosan, and such cells caused hyper-inflammatory responses in mice (Upadhya et al., 2023). This is why we also determined the FA and chitin/chitosan contents of the selected strains WT KN99, *cda1Δ2Δ*, *cda1Δ2Δ3Δ* and *cap59Δ* after cultivation in YNB-pH 7 and YNB-U (Fig. 5). YNB-U strongly increased the cell wall FA compared to YPD (0.93 *vs* 0.62 for WT *Cn*), even more than RPMI conditions. YNB-pH 7 gave inconsistent results, with FA values similar to YPD for WT *Cn* but higher than YPD for *cda1Δ2Δ*. Comparative data are only available for WT *Cn* (Table S1), showing the same strong increase in FA during growth in YNB-U compared to YPD (0.82 *vs* 0.34) but also a considerable increase during growth in YNB-pH 7 compared to YPD (0.56 *vs* 0.34) (Upadhya et al., 2023). The strains showed overall lower chitin/chitosan contents in YNB-pH 7 compared to YPD, but cultivation in YNB-U increased the amount of cell wall chitin/chitosan, especially for *cda1Δ2Δ* and *cda1Δ2Δ3Δ*. In contrast, previous work indicated lower chitin/chitosan levels when WT *Cn* was grown in YNB-U rather than YPD (1.7% *vs* 3.1%) (Upadhya et al., 2023).

Earlier studies considering the cell wall chitosans of WT *Cn* report a wide range of values (Table S1), reflecting the limited accuracy of methods to determine the FA (Upadhya et al., 2021). Enzymatic MS fingerprinting is considered a reliable method for FA analysis because its performance is similar to the gold standard NMR when using pure chitosan samples (Rehman et al., 2023; Wattjes et al., 2020a). However, conventional NMR cannot determine the FA of complex cell wall samples, so we elected to use enzymatic MS fingerprinting for maximum accuracy, sensitivity and reliability (Urs et al., 2023). Our datasets therefore provide a unique comprehensive side-by-side analysis of cell wall chitosans in WT *Cryptococcus* and mutants under various conditions with unprecedented accuracy.

### 2.4 Cell wall chitin/chitosan is more accessible in higher-order mutants and under challenging conditions

**Fig. 6:**
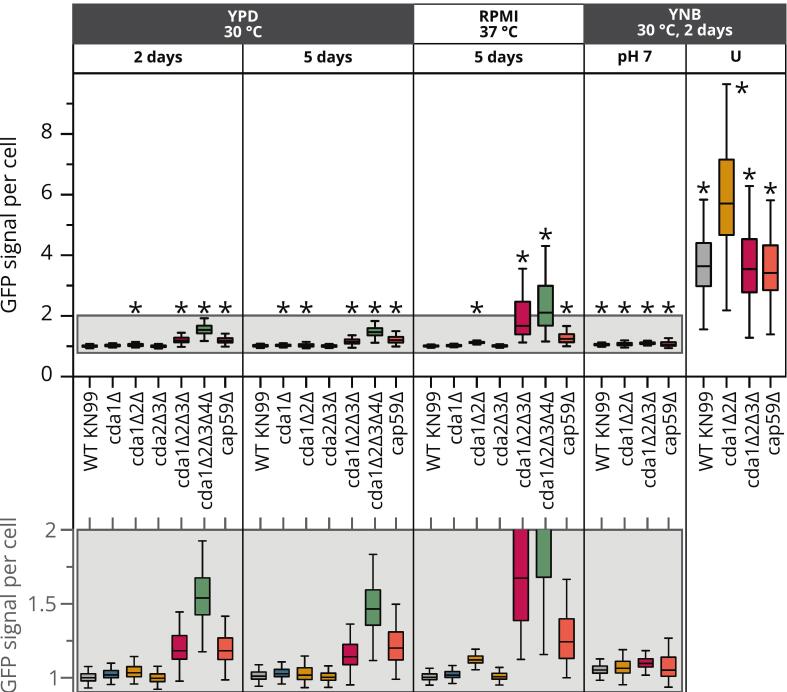
**GFP signal per cell after CBP-sfGFP staining of selected strains grown under different conditions.** The GFP signal per cell was determined for all stained cells in one well of a 96-well plate (*n* = 12,000–67,000, same strain and growth condition per well), and each was divided by the mean GFP signal per cell of the same cells without CBP-sfGFP staining. The lower panel shows a zoom of the samples highlighted with a gray background in the upper panel to visualize small differences in samples with a low GFP signal per cell. The whiskers show the range of data points within 1.5× the interquartile range. Statistical significance (*p < 0.001) was determined by one-way ANOVA with Tukey's multiple comparisons test between each sample and WT KN99 YPD (2 days).

The FA and chitin/chitosan content of *Cryptococcus* cell walls can help to draw conclusions about host–pathogen interactions already, but additional insights are gained by including chitin/chitosan accessibility. Recent solid-state NMR (ssNMR) studies revealed that different carbohydrate polymers in the cryptococcal cell wall are organized into rigid and mobile domains, with well-defined cross-linking among chitin, glucans, and capsule fibers (Ankur et al., 2025). Consequently, when probing the cell wall with molecule-specific reagents, the binding of each probe depends not only on the abundance of the target polymer but also on its accessibility within the overall cell wall architecture. To this end, we stained heat-killed cells of strains grown under different conditions with a chitin-binding protein fused to superfolder GFP (CBP-sfGFP) (Fuenzalida et al., 2014) and used cell imaging to measure the GFP signal per cell (Fig. 6). We expected stronger binding of CBP-sfGFP to cell wall chitin/chitosan with a higher FA because CBP prefers highly acetylated substrates (Fuenzalida et al., 2014). Accordingly, GFP signals per cell were stronger in higher-order mutants where the FA was higher (*cda1Δ2Δ*, *cda1Δ2Δ3Δ* and *cda1Δ2Δ3Δ4Δ*) compared to WT KN99 and mutants with comparably low FA (*cda1Δ* and *cda2Δ3Δ*) (Fig. 4). We also expected stronger binding to more accessible chitin/chitosan, *e.g.*, on the cell wall surface or embedded in a more loosely organized cell wall, because CBP-sfGFP is a much larger molecule than dyes such as calcofluor white or eosin Y, and hence cannot easily penetrate dense matrices (Esher et al., 2018). This was confirmed by the strong GFP signals per cell in the acapsular mutant *cap59Δ* because the capsule can form a dense matrix that restricts diffusion (Doering, 2009; Gates et al., 2004), and in cells grown under challenging conditions (YNB-U or RPMI) leading to altered morphology and compromised cell wall integrity (Upadhya et al., 2023). The higher FAs in higher-order mutants and/or under challenging conditions may also impair cell wall organization and increase chitosan accessibility, thus making it difficult to determine which factor plays a bigger role in governing CBP-sfGFP binding. In addition, more abundant chitin/chitosan provides more binding sites for CBP-sfGFP, resulting in stronger GFP signals per cell. It is difficult to reconcile the strong differences in GFP signals we observed (Fig. 6) with the slight differences in chitin/chitosan content (Fig. 3, Fig. 4, Fig. 5). The stronger signals for strains grown in YNB-U may also partially reflect the increased chitin/chitosan content, not only the improved accessibility due to reorganization of the cell wall as described above.

The quantified data (GFP signal per cell) were consistent with representative images (Fig. S4). No GFP signals were visible in unstained controls, as shown for WT KN99 in YPD (2 days) and *cda1Δ2Δ3Δ* in YNB-U. The intensity of the GFP signal in the stained samples increased in the order WT KN99 in YPD (2 days) < *cda1Δ2Δ3Δ* in YPD (2 days) < *cda1Δ2Δ3Δ* in RPMI < *cda1Δ2Δ3Δ* in YNB-U. Interestingly, the GFP signals for WT KN99 in YPD (2 days) were concentrated at certain points in the cell wall and at the interface between mother and daughter cells during budding. This suggests that the chitosan is more accessible and/or more acetylated at bud necks and bud scars compared to positions where the capsule and cell wall are intact. In contrast, the GFP signals for *cda1Δ2Δ3Δ* were more diffuse: still localized to the cell wall in YPD (2 days) but visible throughout the cells in RPMI and YNB-U, supporting the hypothesis that chitin/chitosan is more exposed in these samples. It will be interesting and certainly revealing to further analyze the cell wall of these mutant strains using the recently emerging ssNMR techniques that allow deep insights into the architecture of the entire cell wall *in situ*, as discussed in more detail below (see section 3).

### 2.5 FA and accessibility of chitin/chitosan influence virulence

**Fig. 7:**
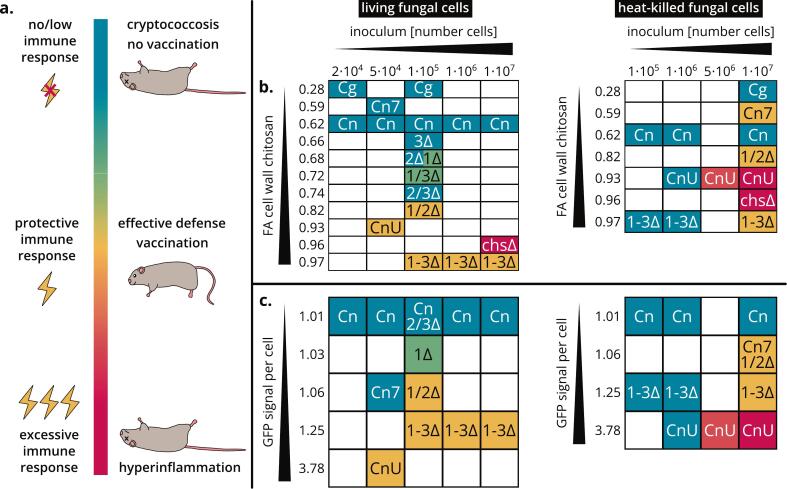
**Models for the relationship between the outcome of fungal infection or vaccination and cell wall FA and accessibility.** (**a**) Three possible outcomes of *Cryptococcus* infection or vaccination according to the damage-response framework of microbial pathogenesis (Casadevall and Pirofski, 2003). (**b**) Relationship between the FA of cell wall chitin/chitosan (Fig. 3, Fig. 4, Fig. 5) and outcome of infection with live *Cryptococcus* cells (left) or vaccination with heat-killed *Cryptococcus* cells (right), color-coded as in panel (a). (**c**) Relationship between GFP signal per cell after CBP-sfGFP staining (Fig. 6) and outcome of infection with live *Cryptococcus* cells (left) or vaccination with heat-killed *Cryptococcus* cells (right), color-coded as in panel (a). Strains and growth conditions are abbreviated as follows and the infection/vaccination data were obtained from the indicated publications. Cn = WT *Cn*, YPD (Hester et al., 2024; Upadhya et al., 2023; Upadhya et al., 2016, Upadhya et al., 2018, Upadhya et al., 2021); Cn7 = WT *Cn*, YNB-pH 7 (Upadhya et al., 2023); CnU = WT *Cn*, YNB-U (Upadhya et al., 2023); 1Δ = *Cn cda1Δ*, YPD (Upadhya et al., 2021, Upadhya et al., 2018); 2Δ = *Cn cda2Δ*, YPD (Upadhya et al., 2021, Upadhya et al., 2018); 3Δ = *Cn cda3Δ*, YPD (Upadhya et al., 2021, Upadhya et al., 2018); 1/2Δ = *cda1Δ2Δ*, YPD (Upadhya et al., 2021); 1/3Δ = *cda1Δ3Δ*, YPD (Upadhya et al., 2021); 2/3Δ = *cda2Δ3Δ*, YPD (Upadhya et al., 2021); 1–3Δ = *cda1Δ2Δ3Δ*, YPD (Hole et al., 2020; Upadhya et al., 2018; Upadhya et al., 2016); chsΔ = *chs3Δ*, YPD (Hole et al., 2020); Cg = WT *Cg* R265 (VGII), YPD (Hester et al., 2024; Lam et al., 2019). More details on the underlying infection/vaccination data are available in the supporting information (sheets “Fig. 7b” and “Fig. 7c” of *Figs. 1–9 + S1–7.xlsx*).

Previous studies suggest that the avirulence of chitosan-deficient *Cryptococcus* mutants is caused by their broken stealth mechanism: chitin can be readily degraded by host chitinases and/or recognized by the host immune system, resulting in a protective immune response (Baker et al., 2011; Hole et al., 2020; Student et al., 2024; Upadhya et al., 2018), as visualized in the scenario of Fig. 7a. For the same reason, chitosan-deficient *Cryptococcus* cells can be used as a vaccine to induce protective immunity against subsequent fungal infections (Hester et al., 2024; Upadhya et al., 2016). According to the damage-response framework of microbial pathogenesis (Casadevall and Pirofski, 2003), disease can result from a host immune response that is either too weak or too strong. In the first case, represented by WT *Cn* grown in YPD, the infection goes undetected and, in the absence of immune defenses, the fungus can proliferate, ultimately leading to severe cryptococcosis (Baker et al., 2011; Hester et al., 2024; Upadhya et al., 2018). Given that these fungal cells do not elicit an immune response, they cannot be used as a vaccine (Upadhya et al., 2023; Upadhya et al., 2016). In the second case, represented by WT *Cn* grown in YNB-U or *chs3Δ*, fungal cells elicit an excessive immune response causing host death by hyper-inflammation (Hole et al., 2020; Upadhya et al., 2023), as observed in IRIS patients (O'Meara et al., 2013; Ost et al., 2017; Perfect, 2012). Fungal cells that trigger hyper-inflammation in humans are also not suitable as a vaccine. It is important to note that the categorization of immune responses as insufficient, adequate or excessive is a simplification that masks gradual transitions. For example, infections with *cda1Δ* and *cda1Δ2Δ* grown in YPD are non-lethal for mice, but *cda1Δ* persists in the lungs and disseminates to the brain, whereas *cda1Δ2Δ* can be cleared by the host (Upadhya et al., 2021; Upadhya et al., 2018).

Based on our data for the different strains and conditions (Fig. 3, Fig. 4, Fig. 5), we attempted to correlate the FA of cell wall chitosan to the outcome of previous mouse infection studies (Fig. 7b, left) or mouse vaccination experiments (Fig. 7b, right) conducted over the last decade, resulting in a hypothetical model to link FA and host immunity. To ensure comparability, the number of fungal cells used as inoculum was taken into account. The results of the published animal experiments were grouped into categories and colored according to the scheme in Fig. 7a. Further details on the underlying *in vivo* data extracted from literature can be found in the supporting information. Mice can effectively defend themselves against infectious strains with higher FAs, whereas low-FA strains lead to cryptococcosis regardless of the inoculum. In vaccination studies, no strains were able to induce protective immunity if the inoculum contained a low number of cells. However, a large number of cells (1 × 10^7^) could trigger any of the three possible outcomes: no protection, protective immunity or hyper-inflammation. Interestingly, already 5 × 10^6^ cells with an FA of 0.93 (WT *Cn* in YNB-U) induced partial hyper-inflammation, but twice as many cells with an even higher FA of 0.97 (*cda1Δ2Δ3Δ*) conferred protective immunity. This implies that FA is not the only factor governing differential immune recognition of the various CDA mutants. We therefore tested whether the GFP signals per cell (Fig. 5) correlated more closely with previous infection (Fig. 7c, left) or vaccination (Fig. 7c, right) studies, because this accounts for both FA and chitin/chitosan accessibility due to altered cell wall architecture, probably in accordance with stronger exposure of other PAMPs as well. This new model linking host immunity to FA and cell wall organization was better than the model based on FA alone (Fig. 7b): the intensity of the immune response (none, protective or excessive) increases with both the number of cells used as inoculum (dose dependency) and the GFP signal per cell.

Previous infection and vaccination studies using *Cg* R265 (VGII) CDA mutants also fit the model of FA-dependent immune responses. First, whereas WT *Cg* R265 (VGII) and CDA mutants with a similar GlcN content (*Cg cda1Δ*, *Cg cda2Δ* and *Cg cda1Δ2Δ*) lead to cryptococcosis, CDA mutants with a lower GlcN content (*Cg cda3Δ*, *Cg cda1Δ3Δ*, *Cg cda2Δ3Δ* and *Cg cda1Δ2Δ3Δ*) are cleared by the host (Lam et al., 2019). Second, *Cg cda1Δ2Δ3Δ* cells are an effective vaccine against subsequent WT *Cg* R265 (VGII) infection (Hester et al., 2024), and induce immune responses in a dose-dependent manner (Lam et al., 2019). Moreover, loss of cell wall integrity or missing layers may be associated with increased exposure of and a more intense host immune response to PAMPs such as chitin, but also mannans, glucans, mannoproteins and other antigens. This has been shown for higher-order CDA mutants, *prm1Δ*, capsule mutants (*mar1Δ*, *rim101Δ*) or strains grown in YNB-U (Esher et al., 2018; Hole et al., 2020; Li et al., 2023; O'Meara et al., 2013; Ost et al., 2017; Upadhya et al., 2023). Based on our data, we cannot state with certainty that it really is the highly acetylated, accessible chitin/chitosans that ultimately trigger immune responses. It is also possible that the impaired cell wall architecture (caused by the higher FA) leads to the exposure of other PAMPs, which activate independent pathways. Even so, the more intense GFP signals per cell can be used to confirm the loss of cell wall integrity and thus the potential exposure of different types of PAMPs.

Overall, we were able to establish a relationship between the outcome of infection or vaccination and the FA and/or accessibility of cell wall chitin/chitosan. Both parameters together, measured by the GFP signal per cell after CBP-sfGFP staining, can be used as an indicator of the strength of the host immune response. Thus, our approach provides the means to easily screen vast libraries of fungal strains for virulence or vaccine effectivity prior to animal experiments. This could help to identify and compare large numbers of strains that do or do not induce an immune response, leading to a better understanding of virulence factors in *Cryptococcus*. Moreover, new hyper-inflammatory strains revealed by this screening could provide insight into the molecular basis of IRIS.

### 2.6 Chitinolytic enzymes impair the growth of higher-order mutants while releasing chitin oligomers

**Fig. 8:**
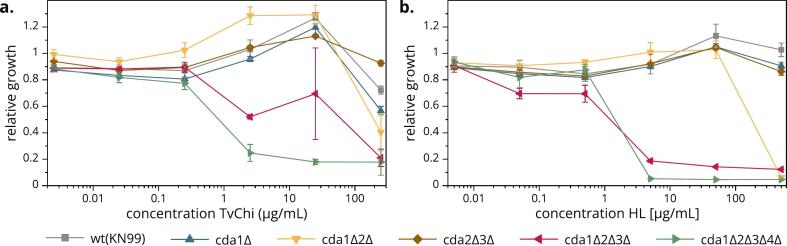
**Growth inhibition of selected *Cn* strains dependent on the concentration of chitinolytic enzymes in the medium.** Shown is the relative growth after 48 h in YNB-U medium supplemented with the indicated amount of (**a**) *Trichoderma virens* chitinase (TvChi) expressed in *E. coli* (Bußwinkel et al., 2018) or (**b**) human lysozyme (HL) expressed in rice (Hellmann et al., 2025). OD_600_ values (Fig. S5) were normalized to the respective control (same strain without added enzyme) to calculate the relative growth. Results of the corresponding unpaired Student's *t*-test can be found in the supporting information (sheet “Fig. 8a” and “Fig. 8b” of *Figs. 1–9 + S1–7.xlsx*). Data are means ± standard deviations of *N* = 3 biological replicates.

Having confirmed a correlation between the host immune response and both the FA and the accessibility of chitin/chitosan in the fungal cell wall, we considered that these polymers are likely degraded *in vivo* by host chitinolytic enzymes. We therefore asked whether differences in FA and chitin/chitosan architecture also affect the susceptibility of fungal cell walls to degradation by host enzymes. First, we tested the influence of chitinolytic enzymes on the growth of different *Cn* strains in YNB-U using the well-characterized TvChi chitinase from *Trichoderma virens* (Bußwinkel et al., 2018) belonging to glycoside hydrolase family 18 (GH18, like CHIT1 and AMCase), as well as HL (GH22). Fig. 8 shows the relative growth of the strains after 48 h plotted against the enzyme concentration in the medium. The corresponding absolute growth and full growth curves over time are shown in Figs. S5–7. TvChi (Fig. 8a) significantly inhibited the growth of WT *Cn*, *cda1Δ* and especially *cda1Δ2Δ*, but only at very high concentrations (250 μg/mL), whereas *cda2Δ3Δ* behaved more similarly to the enzyme-free control. Interestingly, the growth of WT *Cn*, *cda1Δ*, *cda1Δ2Δ* and *cda2Δ3Δ* even improved significantly at high TvChi concentrations (25 μg/mL). In contrast, the growth of chitosan-deficient mutants *cda1Δ2Δ3Δ* and *cda1Δ2Δ3Δ4Δ* was suppressed by TvChi at concentrations as low as 2.5 μg/mL. Similar results were observed for HL (Fig. 8b). The low-FA strains WT *Cn*, *cda1Δ* and *cda2Δ3Δ* did not show strong changes in growth except for a slightly higher OD_600_ at high HL concentrations (50 μg/mL) (only significant for *cda2Δ3Δ*), whereas the intermediate-FA strain *cda1Δ2Δ* was inhibited by very high HL concentrations (500 μg/mL). Both chitosan-deficient mutants failed to grow at HL concentrations ≥5 μg/mL.

The ability of chitinolytic enzymes to inhibit fungal growth *in vitro* has been reported for human CHIT1 against *Mucor rouxii* and *Candida albicans* (van Eijk et al., 2005), and the growth of WT *Cn* was inhibited by cell culture medium containing secreted recombinant CHIT1 (Gordon-Thomson et al., 2009). The latter also reduced mortality in mouse models of neutropenic candidiasis and aspergillosis (van Eijk et al., 2005). Both TvChi and HL act preferentially against highly acetylated substrates (Hellmann et al., 2025), so the cell walls of chitosan-deficient mutants are more susceptible to hydrolysis by these enzymes than WT *Cn*. The loss of cell wall integrity in these mutants (Baker et al., 2007) may also increase the exposure of chitin (Fig. 6), facilitating enzymatic attack. Because *Cn* expresses four GH18 *endo*-chitinases potentially involved in the utilization of chitin from fungal neighbors (Baker et al., 2009), it is conceivable that WT *Cn* has evolved lower susceptibility to extracellular chitinases, including cell wall deacetylation. The higher final OD_600_ in the presence of chitinolytic enzymes matches previous observations that, under conditions such as mild oxidative stress, *Cryptococcus* reacts by increasing growth (Upadhya et al., 2013), probably as a last attempt to spread and find a niche with more favorable conditions.

**Fig. 9:**
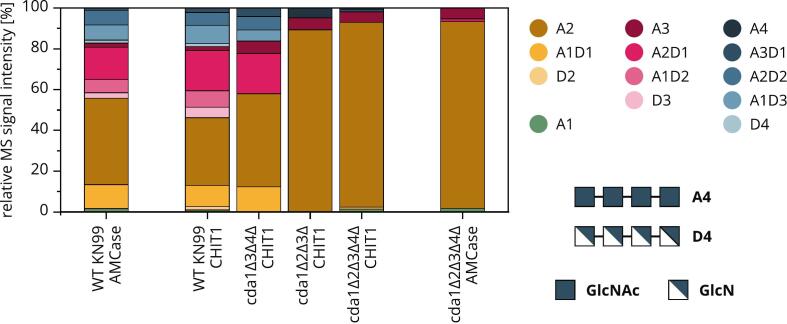
**Product profiles of oligomers released from *Cn* cell walls by CHIT1 or AMCase.** The relative intensities of MS signals representing small products (dimers, trimers and tetramers) released from cell walls of different *Cn* strains cultivated under YPD conditions (2 days) with differences in FA are shown: (WT KN99 = 0.62, *cda1Δ3Δ4Δ* = 0.74, *cda1Δ2Δ3Δ*/*cda1Δ2Δ3Δ4Δ* = 0.97). A represents a GlcNAc unit, and D represents a GlcN unit.

Finally, we compared the products released by CHIT1 or AMCase from the cell walls of WT *Cn*, *cda1Δ3Δ4Δ*, *cda1Δ2Δ3Δ* and *cda1Δ2Δ3Δ4Δ* in YPD (Fig. 9). Consistent with their similar subsite preferences (Hellmann et al., 2025), both human chitinases released the same products when incubated with the same *Cryptococcus* cell walls. We therefore compared the CHIT1 product profiles on *Cn* cell walls with increasing FA (Fig. 9, center) and also detected products with increasing FA, where only chitin oligomers were released from the cell walls of chitosan-deficient strains. The cleavage of cell wall chitin/chitosan by host enzymes may not only be a direct weapon against fungal pathogens but the released oligomers may alert the host immune system by binding to PRRs such as TLR2 (Chang et al., 2022; Da Silva et al., 2009, Da Silva et al., 2008; Elieh Ali Komi et al., 2018; Feng et al., 2004). Here, not only the amount, but also the length, FA and PA of the released oligomers seem to control the magnitude of the immune response (Fuchs et al., 2018; Gorzelanny et al., 2010; Hembach et al., 2020). *Cn* also produces chitinases (Baker et al., 2009) with the potential to interfere with host immunity based on chitinous oligomers released by host enzymes. The enzymatic degradability and product profiles add more layers to the already complex picture of the interaction between *Cryptococcus* chitin/chitosan and the host. Ultimately, these aspects contribute to the ability of the host to detect the fungus, trigger appropriate immune responses, and mount an effective defense.

## 3 Conclusion and outlook

We have presented the first comprehensive side-by-side analysis of cell wall chitin and chitosan in various *Cryptococcus* strains under different growth conditions, comparing growth, FA, chitin/chitosan content and accessibility, susceptibility to chitinolytic enzymes, and enzyme product profiles. The importance of CDA1 for the conversion of cell wall chitin to chitosan was confirmed, especially under challenging conditions (Upadhya et al., 2018). Future promoter exchange studies should address whether CDA1 is necessary because it is the most abundant or because it has a unique activity, such as the production of chitosans with a specific PA. CDA2 has been shown to support cell wall deacetylation by CDA1 during infection, possibly acting on the products of CDA1 as preferred substrates, whereas CDA3 plays a minor role (Upadhya et al., 2021). In support of this, our knockout of both *CDA1* and *CDA2* was always accompanied by the most substantial changes in cell wall FA. The challenging conditions (including host-mimicking conditions) reduced cell wall deacetylation in general, which was surprising given that low cell wall FA seems to be a necessary part of the stealth mechanism to avoid host immune detection. Survival at elevated temperatures (37 °C) with a suboptimal nutrient supply may require a certain degree of cell wall rigidity, and *Cryptococcus* may therefore make a trade-off between stealth (lower FA) and the stress tolerance conferred by rigidity (higher FA). However, a fully acetylated cell wall appears to be detrimental to both (Baker et al., 2007). Such trade-offs seem likely when considering *Cryptococcus* as an environmental fungus and human pathogen, which must therefore deal with the challenges of both worlds and may have acquired its virulence during its non-pathogen lifestyle in the ‘environmental virulence school’ (Lange et al., 2023).

**Fig. 10:**
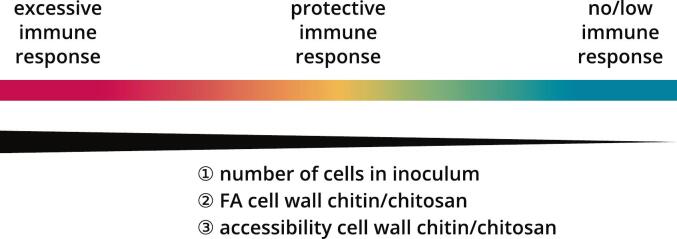
**Overview of established correlations between strength of host immune response and inoculum, cell wall FA, or chitin/chitosan accessibility of the cryptococcal cells.** The color scheme corresponds to the one in Fig. 7, the respective data leading to these conclusions are presented in Fig. 7b and c as well as in the references cited in the legend to Fig. 7. An excessive immune response leads to potentially fatal hyper-inflammation in the host. A protective immune response either leads to an effective defense in case of living fungal cells, or to an effective vaccination against subsequent infection in case of heat-killed fungal cells. If the immune response is low or absent, living fungal cells cause cryptococcosis while heat-killed fungal cells are unsuitable for vaccination.

We applied the damage response framework of microbial pathogenesis (Casadevall and Pirofski, 2003) to *Cryptococcus*, creating a model that links inoculum as well as both FA and chitin/chitosan accessibility to the strength of the host immune response and corresponding outcomes of infection (cryptococcosis, effective defense, or hyper-inflammation) (Fig. 10). Accordingly, our methods for cell wall FA analysis and especially CBP-sfGFP staining are valuable tools for future studies on immunity, virulence, disease, and vaccination. For example, large strain libraries could be screened for high CBP-sfGFP signals to identify candidates which potentially induce strong immune responses in subsequent animal experiments. In the future, the influence of chitin/chitosan content, FA and accessibility on proliferation, enzymatic degradation, receptor binding and signaling, phagocytosis, and intracellular survival should be studied in more detail.

A better understanding of the mode of action of fungal CDAs is also needed to obtain a complete picture of host–pathogen interactions. Although CDAs 1–4 have been identified and expressed as recombinant proteins, they have mainly been tested for their immunoeliciting properties (Biondo et al., 2005; Biondo et al., 2002; Levitz et al., 2001; Levitz and Specht, 2006; Specht et al., 2022; Specht et al., 2007, Specht et al., 2017; Wang et al., 2023). Their behavior as enzymes *in vitro*, such as mode of action on oligomers and/or polymers, has only been tested for CDA2 and CDA4 (Hembach et al., 2020, 2017; Sreekumar et al., 2022). These studies revealed differences between the enzymes (Hembach et al., 2017) and an unusual preference for GlcN units at subsite −1 of CDA4, leading to its identification as a chitosan deacetylase and its proposed role as a security guard (Hembach et al., 2020). The characterization of CDA1 and CDA3, and further *in vitro* analysis of CDA2 and CDA4 according to recently published guidelines (Lindner et al., 2025) is required to understand how similarities and differences between these enzymes affect their biological roles, and why *Cryptococcus* needs four distinct CDAs. Unexpectedly, the triple mutant *cda1Δ2Δ3Δ* consistently grew more slowly than all other *Cn* strains, including the quadruple mutant *cda1Δ2Δ3Δ4Δ* (Figs. 2 and S5). To confirm that the presence of CDA4 in the absence of all other CDAs is indeed responsible for this difference, we would need to analyze a new *cda1Δ2Δ3Δ* mutant (by introducing the *CDA4* gene into the existing *cda1Δ2Δ3Δ4Δ* strain) or *cda1Δ2Δ3Δ4Δ* mutant (by knocking out *CDA4* in the existing *cda1Δ2Δ3Δ* strain).

Notably, our reported FA values are averages of all chitinous cell wall components, and therefore do not provide any information about FA dispersity. Accordingly, WT *Cn* cell walls could contain a mixture of fully acetylated chitin and highly deacetylated chitosans, or only chitosans of an intermediate FA, or anything between these extremes. Analysis of FA dispersity is challenging even for pure chitosan samples (Wattjes et al., 2020b) and has never been reported for biological materials such as fungal cell walls. Even so, this aspect should not be neglected, because it strongly influences the binding of receptors and enzymes, product profiles, the flexibility and charge distribution of the cell wall, and finally the outcome of host immune responses.

Finally, the precise cell wall architecture, including the order of layers and their spacing and thickness, should be analyzed in detail (Diniz-Lima et al., 2022; Garcia-Rubio et al., 2020; He et al., 2024; Iyer et al., 2021; Sato and Kawakami, 2022) because our data indicate the importance of access to cell wall components. In the past, chemical analysis of cell wall components required their prior extraction from the cell wall, potentially altering sensitive structural details and limiting information on molecular interactions of cell wall components and their distribution with the different cell wall layers. However, novel biophysical methods, especially ssNMR, open up possibilities to study intact cell walls *in situ*, thus potentially revolutionizing our understanding of fungal cell wall architectures (Latgé and Wang, 2022). Recent ssNMR studies of mushrooms and ascomycetous or zygomycetous fungi have provided unprecedented insights into cell wall composition, organization, interactions, and dynamics (Cheng et al., 2024; Fernando et al., 2021; Kang et al., 2018; Safeer et al., 2023). The first ssNMR analyses of *Cn* cell walls revealed, *i.a.*, interconnectedness between the different cell wall polysaccharides, a large number of different chitin allomorphs, and strong dynamics of the capsule glycans (Ankur et al., 2025; Lends et al., 2025). For *Aspergillus fumigatus*, the novel methods showed that stress and mutations in the cell wall machinery lead to strongly altered cell wall organization and activation of compensatory mechanisms (Latgé and Wang, 2022). Performing similar biophysical *in situ* studies on WT *Cn* and CDA mutants cultivated under different conditions will undoubtedly provide a larger picture how the increased FA alters the organization of the entire cell wall, potentially leading to stronger exposure of PAMPs, including chitin.

A greater understanding of chitin and chitosan in host–pathogen interactions will also allow us to extend the analysis to include other cell wall components, such as the capsule, α-glucans, β-glucans, proteins, and melanin (Goodridge et al., 2009; Mukaremera, 2023). Even without these additional factors, the results of our study support the hypothesis that CDA-catalyzed chitin-to-chitosan conversion is not only an integral part of the interaction between fungi and plants (Cord-Landwehr et al., 2016; Student et al., 2024), but also between fungi and mammals (Upadhya et al., 2018). The production of chitosans with fine-tuned FA and PA, forming a less immunoreactive layer to shield PAMPs and/or inhibit degradation by chitinases that release immunoreactive oligomers, may indeed represent a transkingdom virulence strategy (Chang et al., 2022; Lam et al., 2019). Understanding the underlying molecular basis of this strategy will help to elucidate the components of cryptococcal virulence, and in turn facilitate the development of effective treatments or vaccines against this dangerous human pathogen.

## 4 Materials and methods

### 4.1 Cultivation of fungal strains

For cell wall preparation and isolation, *Cryptococcus neoformans* WT KN99α (VNI) and mutants (CDA knockouts, *chs3Δ* CHCN1221 and *cap59Δ* LBCN420) (Baker et al., 2007; Hole et al., 2020; Upadhya et al., 2024), WT *Cryptococcus gattii* R265 (VGII) (Upadhya et al., 2016), and WT *Saccharomyces cerevisiae* BY4741 (ATCC 4040002) were cultivated under various conditions. Strains cultivated in YPD broth (1% yeast extract, 2% Bacto peptone, 2% dextrose), YNB-U (0.67% yeast nitrogen base without amino acids (#291940; Difco) containing 2% dextrose), or YNB-pH 7 (YNB medium adjusted to pH 7 with 50 mM 3-morpholinopropane-1-sulfonic acid (MOPS)) were grown in 50 or 60 mL of medium (YNB or YPD, respectively) in 250-mL polycarbonate Erlenmeyer flasks (Corning, USA) at 30 °C, shaking at 300 rpm. Cells grown on YPD agar plates at 30 °C were used for inoculation. The OD_600_ was measured in a flat-bottom 96-well plate (Corning) containing 200 μL of a 1:5 or 1:10 dilution (YNB or YPD, respectively) of the culture in phosphate-buffered saline (PBS). For the YPD samples, 25 mL of the culture was harvested after 48 h (YPD 2 days). The remaining 35 mL was cultivated further, and another 25 mL was harvested after 120 h (YPD 5 days). For the YNB-pH 7 and YNB-U samples, the entire 50 mL culture was harvested after 48 h. The harvested cell suspension was collected in a 50-mL Falcon tube, followed by centrifugation (3220 ×*g*, 10 °C, 8 min), and the cells were washed twice with cold PBS (50 mL in the first step, 25 mL in the second step). Finally, the cells were resuspended in 5 mL PBS, 2 mL of which was transferred to a 2-mL screw-cap tube (BioSpec Products, USA) for cell wall extraction, and 400 μL to second tube to prepare heat-killed cells. For samples with limited growth (*cda1Δ2Δ3Δ* in YNB-pH 7 or YNB-U), the cells were resuspended in 1–1.5 mL PBS, 200 μL of which was transferred to a screw-cap tube and mixed with 200 μL PBS to prepare heat-killed cells, and the remaining liquid was transferred to another screw-cap tube for cell wall extraction.

To mimic host conditions, strains were cultivated in 60 mL RPMI 1640 medium (#10–040-CM; Corning), containing 10% fetal bovine serum (FBS; #26140; Gibco, Thermo Fisher Scientific, USA) in a triple-layer cell culture flask (500 cm^2^ total culture area, non-treated surface, filter lid; Thermo Fisher Scientific) with 20 mL medium per layer in a 5% CO_2_ atmosphere at 37 °C without agitation. Approximately 3 × 10^7^ cells grown in YPD for 2 days and washed with PBS were used to inoculate each cell culture flask. The OD_600_ was measured in a flat-bottom 96-well plate containing 200 μL of a 1:4 dilution of the culture in PBS. The entire 60-mL culture was harvested after 120 h. The flask was agitated, and 50 mL of the culture was poured into a 50-mL Falcon tube, which was centrifuged (3220 ×*g*, 10 °C, 8 min), followed by decanting the supernatant. The flask was rinsed with 40 mL PBS, and the remaining liquid was added to the pellet in the Falcon tube. After centrifugation as above, the supernatant was removed and the loose cell pellet was washed twice with cold PBS (50 mL in the first step, 25 mL in the second step). The washed cells were resuspended in the remaining PBS after decanting (1–1.5 mL), 200 μL of which was transferred to a screw-cap tube and mixed with 200 μL PBS to prepare heat-killed cells, and the remaining liquid was transferred to another screw-cap tube for cell wall extraction.

To investigate the influence of temperature, medium and pH on growth in more detail (Fig. S2), some of the aforementioned strains were cultivated in 4 mL YPD, RPMI, YNB-pH 7 or YNB-U in one-layer cell culture flasks (25 cm^2^ culture area, non-treated surface, filter lid; Becton Dickinson, USA) at 30 °C, 37 °C, or 37 °C in a 5% CO_2_ atmosphere, always without agitation. The OD_600_ was measured in a flat-bottom 96-well plate containing 200 μL of the non-diluted culture after 24, 50 and 75 h. After 75 h, the liquid was transferred to a 24-well plate, which was centrifuged (2250 ×*g*, 10 °C, 8 min) before measuring the pH of the supernatant.

### 4.2 NF-κB-SEAP reporter cell assay

RAW-Blue cells (InvivoGen, USA) were cultured and passaged according to the manufacturer's protocol before seeding 1 × 10^6^ cells per well into a 96-well flat-bottom plate. WT and mutant yeast strains were grown in 50 mL of the appropriate medium for 48 h at 30 °C, and harvested by centrifugation (3220 ×*g*, 4 °C,10 min) before washing three times with endotoxin-free PBS (#TMS-012 A; EMD Millipore, USA). The final cell suspension for presentation to macrophages was prepared in RPMI 1640 medium containing 10% FBS.

Yeast cells were added to the macrophages at a multiplicity of infection of 20. The yeast–macrophage co-cultures were incubated for 24 h with occasional mixing. Following incubation, 20 μL of culture supernatant was collected to measure SEAP activity using Quanti-Blue (InvivoGen) as the substrate. The supernatant was incubated with Quanti-Blue for 2–8 h at 37 °C, and absorbance was measured at 630 nm.

### 4.3 Isolation of cell walls

The harvested and washed cells in screw-cap tubes were sedimented by centrifugation (15,294 ×*g*, 4 °C, 10 min), the supernatant was removed, and the cell pellet was frozen and lyophilized. We added ∼0.5 mL zirconia beads (0.5 mm diameter; BioSpec Products) and the dried cell pellet was disrupted in a Mini-Beadbeater-96 (BioSpec Products) in a cold aluminum rack (−80 °C) for 2 × 2 min at 2400 strokes/min, with a 2-min break. We added 1 mL of RIPA buffer (100 mM Tris-HCl pH 7.5, 150 mM NaCl, 1% (*w*/*v*) sodium deoxycholate, 1% (*v*/v) Triton X-100, 0.1% (w/v) sodium dodecylsulfate) to the powdered material and beads, followed by incubation with constant rotation for 1 h, and bead beating for 10 s at 2400 strokes/min. After the beads had settled, the liquid was transferred to a 15-mL Falcon tube. The remaining beads were rinsed twice with 1 mL RIPA buffer, and the liquid was transferred to the 15-mL Falcon tube. The isolated cell wall material was rinsed three times by centrifugation (3220 ×*g*, 10 °C, 8 min), followed by removal of the supernatant, and resuspension of the pellet in 15 mL PBS. A fourth rinsing step was performed in the same way, but the pellet was resuspended in 1 mL PBS. The sample was transferred from the Falcon tube to a screw-cap tube. The Falcon tube was then rinsed with 0.5 mL PBS, which was added to the sample in the screw-cap tube. Finally, the screw-cap tubes were centrifuged (15,294 ×*g*, 10 °C, 8 min), the supernatant was removed, and the pellet was frozen and lyophilized.

### 4.4 Determination of cell wall chitin/chitosan content and FA

Cell wall preparations were analyzed as previously described (Urs et al., 2023). Briefly, 1 mg of lyophilized cell wall material was treated with KOH, rinsed, and GlcN units were chemically *N*-acetylated with [^2^H_6_]-acetic anhydride. The cell pellet was washed and treated with an enzyme cocktail consisting of a chitosanase from *Streptomyces sp.* N174 (Weikert et al., 2017), and chitinases from *Trichoderma viride* (Sigma-Aldrich, USA) and *Serratia marcescens* (Sørbotten et al., 2005) to fully hydrolyze the chitin to GlcNAc monomers (with or without the [^2^H_3_] label). The samples were analyzed by ultra-high-performance liquid chromatography electrospray ionization mass spectrometry (UHPLC-ESI-MS). The intensities of the GlcNAc monomer signals with or without [^2^H_3_] were used to calculate the FA. For the absolute quantification of GlcNAc monomers with or without [^2^H_3_], these signals were compared to a defined amount of double isotope-labeled internal standard ([^13^C_2_, ^2^H_3_] GlcNAc).

### 4.5 CPB-sfGFP staining and imaging

The 400 μL cell suspension in a screw-cap tube prepared as described above (see section 4.1) was heated to 70 °C for 20 min to kill the cells, and then diluted with PBS to an OD_600_ of ∼0.5. We transferred 100 μL of the diluted suspension to a round-bottomed 96-well plate, which was centrifuged (2250 ×*g*, 10 °C, 10 min) and flipped to remove the supernatant. The cells were resuspended in 100 μL PBS with 3% (w/v) bovine serum albumin, and the sample was split: 50 μL was transferred to a fresh round-bottomed 96-well plate for staining, and the remaining 50 μL was retained as an unstained control. To stain the cells, 50 μL of a solution of CBP-sfGFP (Fuenzalida et al., 2014) (0.2 g/L in PBS) was added to the 50 μL cell suspension. For the unstained control, 50 μL of PBS was added instead. In each case, the round-bottomed 96-well plate was incubated in the dark at room temperature shaking at 800 rpm for 1 h, after which the staining solution or PBS was removed by centrifugation (2250 ×*g*, 10 °C, 10 min), the plate was flipped to remove the supernatant, and the cells were resuspended in 200 μL PBS. The exchange of PBS was repeated twice to finally obtain a cell suspension in PBS with an OD_600_ of ∼0.125. This stock suspension was then diluted 1:2.5, 1:10 or 1:25 with PBS (OD_600_ = 0.05, 0.0125 or 0.005, respectively) in a total volume of 200 μL in a flat-bottomed 96-well plate for imaging on an ImageXpress Pico Automated Cell Imaging System (Molecular Devices, USA).

Images were initially captured using the transmitted light (TL) channel, followed by the fluorescein isothiocyanate (FITC) channel. Each sample (combination of strain and growth conditions) was measured with and without CBP-sfGFP staining in all three dilutions and imaged at three magnifications (10×, 20× and 40×). For each magnification, nine images were captured per well, corresponding to total imaged areas of 15.05, 3.76 and 0.94 mm^2^ per well for 10×, 20× and 40×, respectively. Surface detection was achieved in well bottom mode, and the digital confocal was enabled for the FITC channel. To ensure comparability, the exposure time in the FITC channel was consistently set to 250 ms. For the TL channel, the exposure time was adjusted to the corresponding magnification (17.85, 65 and 280 ms for 10×, 20× and 40×, respectively).

Images were analyzed using MetaXpress (Molecular Devices). The cells were identified in the TL channel based on their size and shape (Bottom Hat filter with pixel size 10, filter shape = circle, and activated grayscale reconstruction) as well as their contrast relative to background (HBasin filter with threshold 1000), and holes were filled (FillDarkHoles) to ensure detection of the entire cell area. The output mask was then refined by applying the methods FindRoundObjects (minimum 3.5 μm, maximum 25 μm, intensity 100) and FilterMask (standard area count minimum 1, average intensity minimum 550, area minimum 5), leading to a final mask that successfully differentiated between cells and background, and that could identify individual cells. To quantify the GFP signal per cell, the mask was applied to the FITC channel (standard area 22, no object overlay) and the GFP intensity per cell and cell count per well were exported. Images at 10× magnification captured more cells while still maintaining high-quality data processing and were therefore used to determine the GFP signal per cell, whereas depicted images are at 40× magnification (Fig. S4). To avoid the 10× magnification data differing substantially in the number of cells imaged per sample (combination of strain and growth conditions), we only used data from the dilution (OD_600_ = 0.05, 0.0125 or 0.005) that fitted best into the overall range of the number of imaged cells (final range 12,000–67,000 imaged cells per sample). To normalize the stained sample to the unstained control, the GFP signal per cell was determined individually for each CBP-sfGFP-stained cell, and this value was divided by the mean GFP signal per cell of the same cells without CBP-sfGFP staining.

### 4.6 Growth experiments in the presence of chitinolytic enzymes

Selected strains were cultivated in tilted 14-mL snap-cap tubes (Corning) containing 4 mL YPD at 30 °C shaking at 300 rpm overnight. Cells were harvested by centrifugation (3220 ×*g*, 10 °C, 8 min), washed with 10 mL PBS, and resuspended in 10 mL fresh PBS. The OD_600_ of this stock suspension was determined in a flat-bottom 96-well plate containing 200 μL of a 1:10 dilution of the stock with PBS. The stock suspension was diluted with YNB-U to OD_600_ = 6.25 × 10^−4^ and stored on ice. Stock solutions of *Trichoderma virens* chitinase (TvChi, expressed in *Escherichia coli*) (Bußwinkel et al., 2018) and HL (expressed in rice; Sigma-Aldrich) (Hellmann et al., 2025) were prepared at 20-fold the final concentrations, the latter ranging from 0.0025 to 250 μg/mL for TvChi and from 0.005 to 500 μg/mL for HL. We then transferred 10 μL of each enzyme stock solution (or 10 μL water for the enzyme-free control) to a flat-bottomed 96-well plate (Costar, clear; Corning), and added 190 μL of the cold stock cell suspension (OD_600_ = 6.25 × 10^−4^). Each sample (or control) was prepared in triplicate, and each plate included a minimum of three controls with neither enzyme nor cells (10 μL water +190 μL YNB-U). The plate was sealed with a lid and incubated at 30 °C without agitation in a SpectraMax iD3 spectrophotometer (Molecular Devices). The OD_600_ was measured every 15 min for 48 h to follow the growth over time. The average OD_600_ values of the different samples were compared at the end point after 48 h.

### 4.7 Analysis of CHIT1 or AMCase products on cell walls

We soaked 500 μg of isolated dry cell walls from selected mutants in 75 mM ammonium acetate buffer (pH 5) at room temperature, shaking at 500 rpm overnight. We then added 250 ng of CHIT1 or AMCase (produced in HEK293 cells) (Hellmann et al., 2025), followed by incubation at 37 °C for 24 h, shaking at 500 rpm. The samples were passed through nylon centrifugal filters with a 0.2 μm pore size (VWR International, USA) and analyzed by size exclusion chromatography with refractive index detection and mass spectrometry (Hellmann et al., 2024). Data were analyzed using DataAnalysis v4.1 (Bruker) and an in-house Python script based on the module pymzML (Kösters et al., 2018).

## CRediT authorship contribution statement

**Margareta J. Hellmann:** Writing – review & editing, Writing – original draft, Visualization, Supervision, Methodology, Investigation, Funding acquisition, Formal analysis, Conceptualization. **Rajendra Upadhya:** Writing – review & editing, Supervision, Methodology, Investigation, Formal analysis. **Evelyn Tchoub:** Writing – review & editing, Investigation. **Bruno M. Moerschbacher:** Writing – review & editing, Supervision, Resources, Funding acquisition, Conceptualization. **Jennifer K. Lodge:** Writing – review & editing, Supervision, Resources. **Stefan Cord-Landwehr:** Writing – review & editing, Supervision, Methodology, Conceptualization.

## Funding

Margareta J. Hellmann would like to thank the German Academic Scholarship Foundation (Studienstiftung des Deutschen Volkes) for her doctoral scholarship. This work was funded by the Deutsche Forschungsgemeinschaft (DFG, German Research Foundation) – project numbers 524352358 and 525934838 in the framework of the priority program SPP 2416 “CodeChi” (https://codechi.de) and supported by NIH grant AI125045 to Jennifer K. Lodge.

## Declaration of competing interest

The authors declare that they have no known competing financial interests or personal relationships that could have appeared to influence the work reported in this paper.

## Data Availability

The raw data of the figures are available as part of the supplementary information. Any further details will be made available on request.

## Appendix A Supplementary data

Supplementary material

Figs. 1-9 + S1-7.xlsx
