# Supplementary material for: Acetylation and accessibility of Cryptococcus neoformans cell wall chitosans influence the strength of host immune responses
Source: Cell Surf. 2026 May 20;15:100175. doi: 10.1016/j.tcsw.2026.100175 (PMC13217601; doi:10.1016/j.tcsw.2026.100175)
Supplement: Supplementary file 1 — Supplementary material [file mmc1.pdf]

Supporting information

Acetylation and accessibility of *Cryptococcus neoformans* cell wall chitosans influence the strength of host immune responses

This PDF file includes:

Figures S1 to S7
Table S1
SI References

Other supporting materials for this manuscript include the following:

Table S1 in xlsx format
raw data for Figures 1-9 + S1-7 in xlsx format

Figures

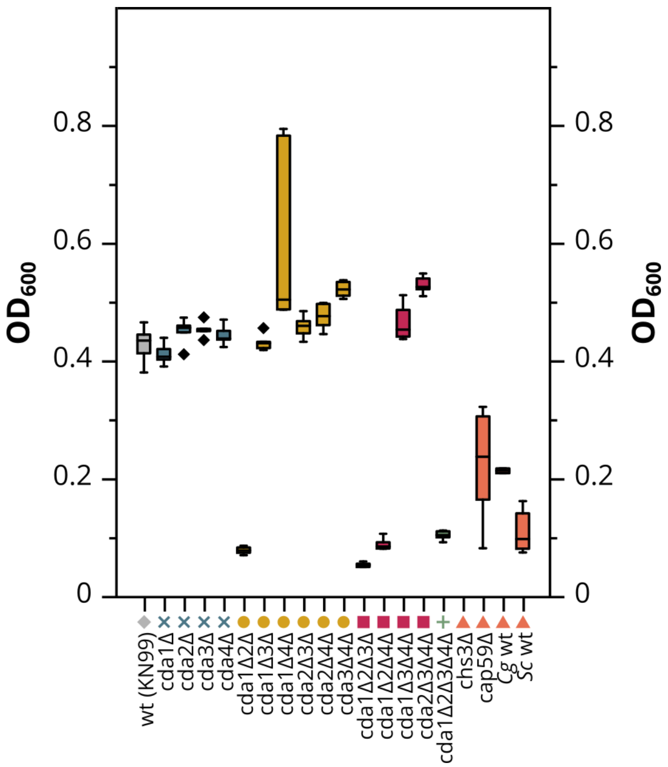

Fig. S1. Growth of *Cryptococcus* strains under RPMI conditions (5 days, 37 °C). The plot shows the RPMI panel of Figure 2 in more detail. For each *Cn* strain, biological triplicates were cultivated (N = 3) and the OD_{600} was measured in technical duplicates for each (n = 6). For WT *Cg* R265 (VGII), only one biological replicate was cultivated and measured as a technical duplicate (n = 2). The whiskers show the range of data points within 1.5× the interquartile range.

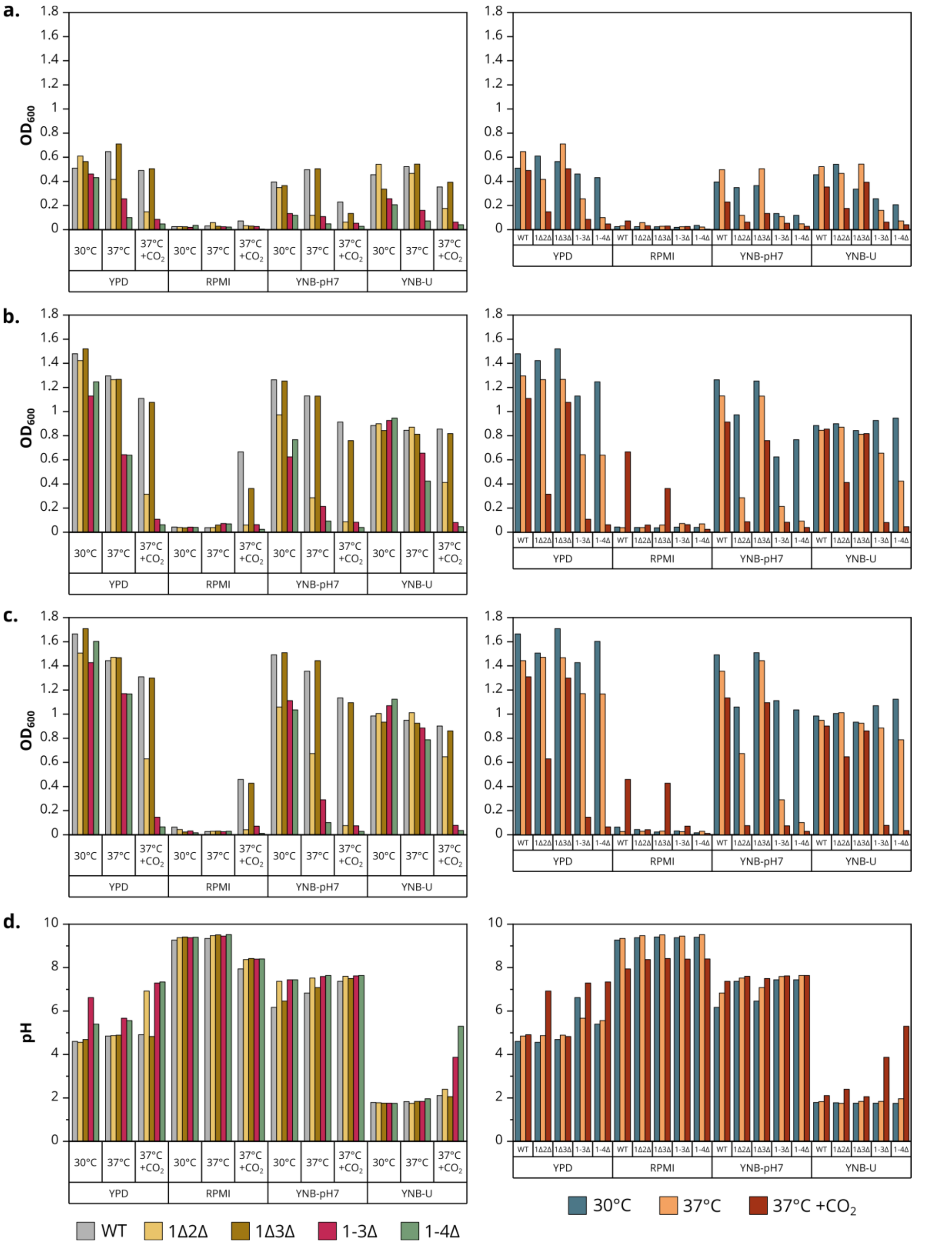

Fig. S2. Growth of *Cryptococcus* strains under different conditions. Cells were cultivated for (**a**) 24 h, (**b**) 50 h and (**c**) 75 h before measuring the OD_{600}. After 75 h, the pH of the medium was also measured (**d**). Left and right panels show the same data but grouped by medium and temperature (left) or by medium and mutant (right). For each strain and condition, the OD_{600} and pH were measured once (n = 1). The CDA mutants are abbreviated as follows: *cda1Δ2Δ* (1Δ2Δ), *cda1Δ3Δ* (1Δ3Δ), *cda1Δ2Δ3Δ* (1-3Δ) and *cda1Δ2Δ3Δ4Δ* (1-4Δ).

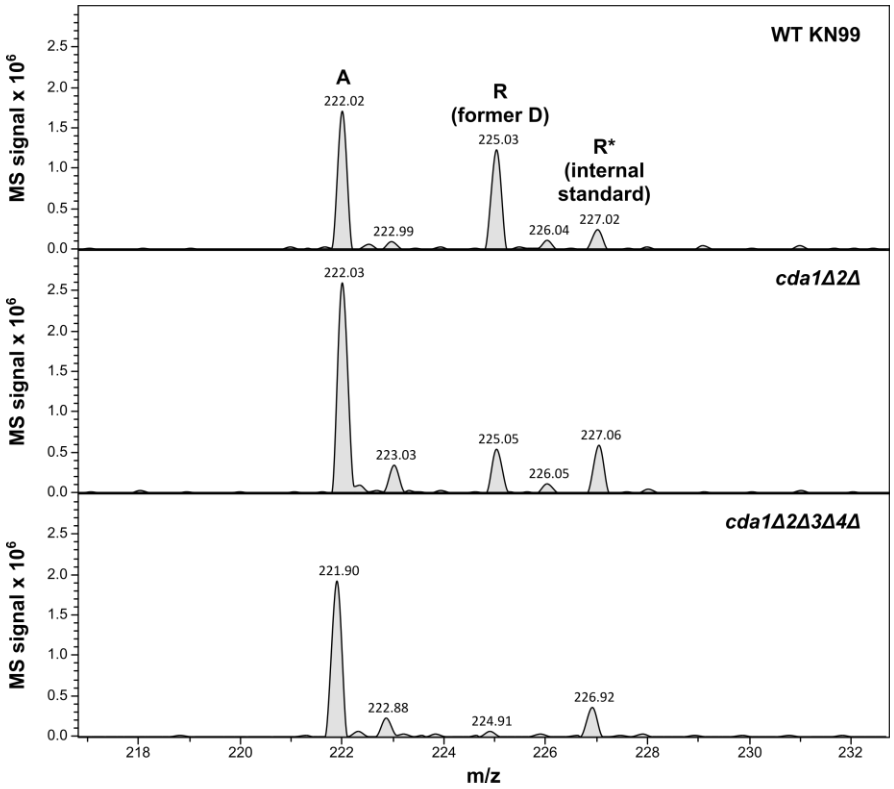

Fig. S3. Representative mass spectra of chitosan monomers and the internal standard. After fungal cell walls were fully hydrolyzed enzymatically into monomers, the latter were measured by UHPLC-ESI-MS. The height of the R* peak corresponds to 50 ng internal standard and can be used to determine the amounts of A and R, which in turn allow the calculation of the FA and chitin/chitosan mass fraction. A = GlcNAc, R = [$^{2}H_{3}$] GlcNAc, D = GlcN, R* = [$^{13}C_{2}$,$^{2}H_{3}$] GlcNAc.

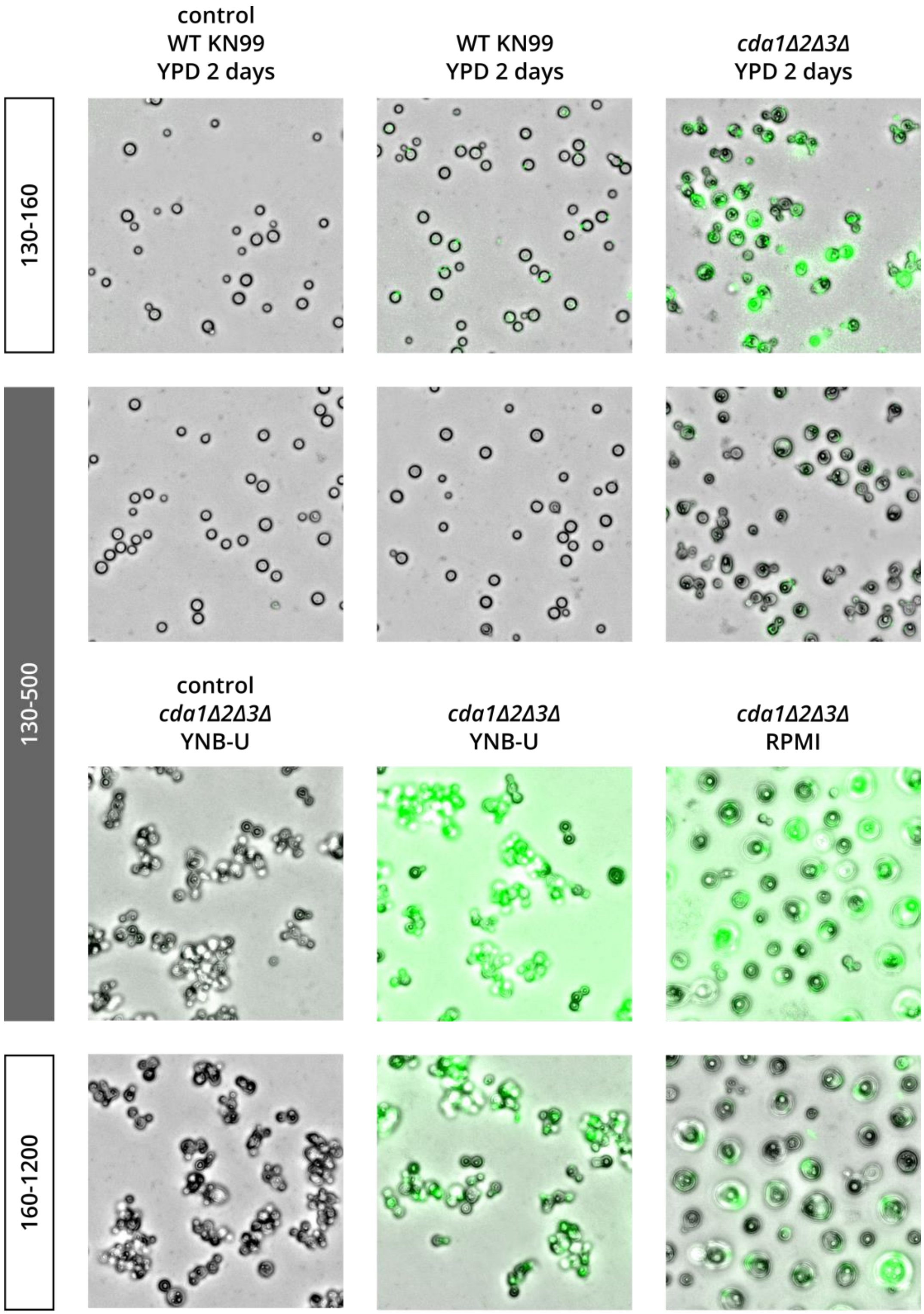

Fig. S4. Representative cell images after CBP-sfGFP staining (40x magnification). The images are composites of the TL channel (black and white) and FITC channel (green), with the GFP signal intensity visible in the latter. Brightness and contrast settings are consistent for all TL images (minimum 1407, maximum 2170) but were varied for the FITC images to optimize the visibility of weak, medium or intense GFP signals, as indicated (130–160, 130–500 and 160–1200, respectively). Comparisons of GFP signals should therefore only be made between images with the same settings. Controls show cells imaged without CBP-sfGFP staining.

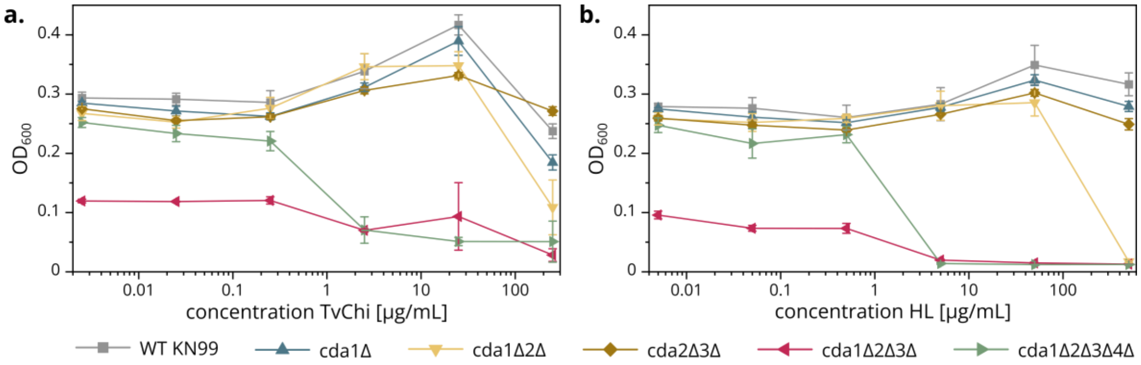

Fig. S5. Growth inhibition of selected *Cn* strains dependent on the concentration of chitinolytic enzymes in the medium. OD_{600} values are shown after 48 h in YNB-U medium supplemented with the indicated amount of (**a**) *Trichoderma virens* chitinase (TvChi) expressed in *E. coli* (Bußwinkel et al., 2018) or (**b**) human lysozyme (HL) expressed in rice (Hellmann et al., 2025). Data are means ± standard deviations of N = 3 biological replicates.

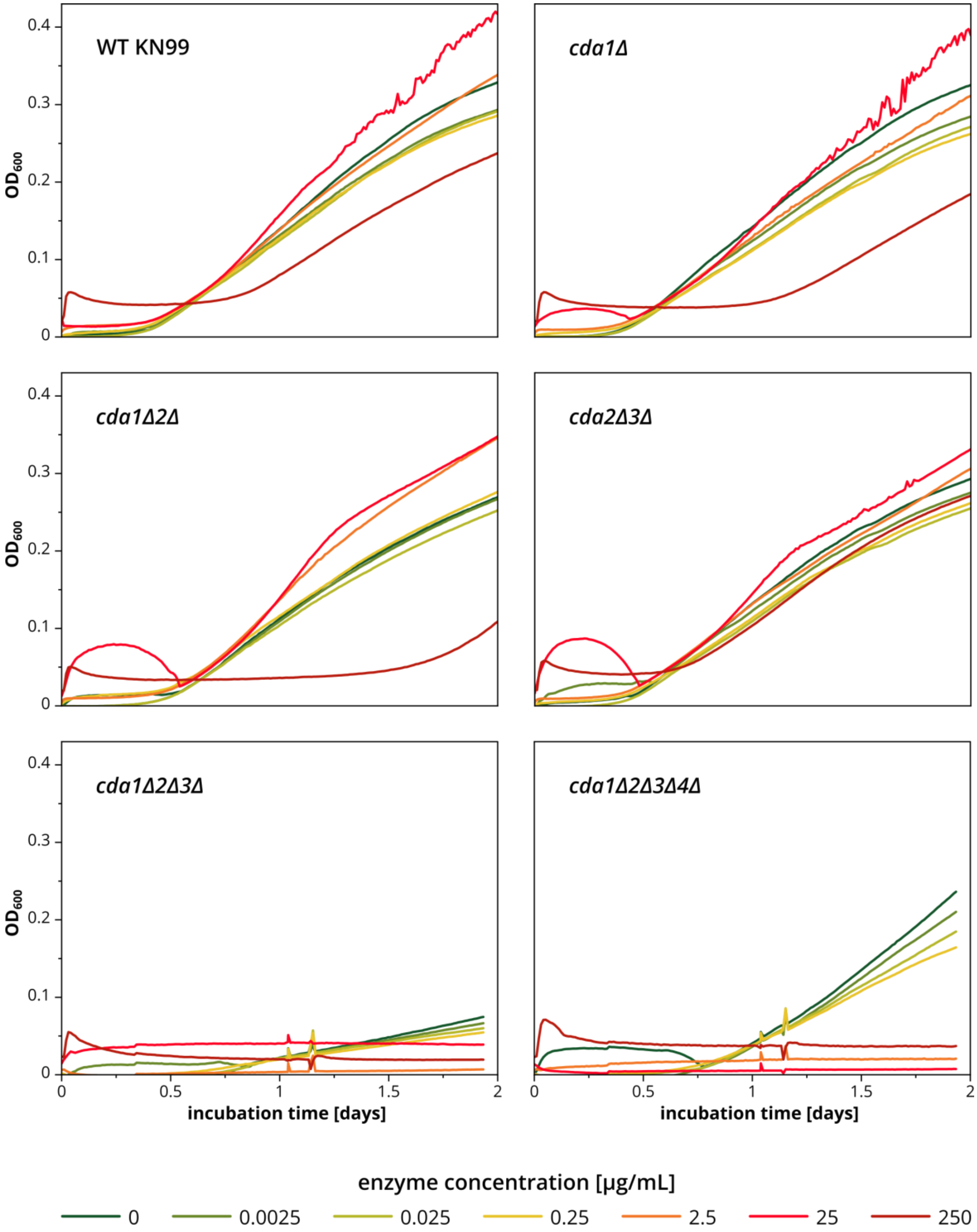

Fig. S6. Growth of selected strains over time in the presence of *Trichoderma virens* chitinase (TvChi). The indicated strains were cultivated in a 96-well plate in YNB-U medium at 30 °C in a spectrophotometer, and the OD_{600} was measured every 15 min. The different curves correspond to different concentrations of TvChi (Bußwinkel et al., 2018) and are means of N = 3 biological replicates.

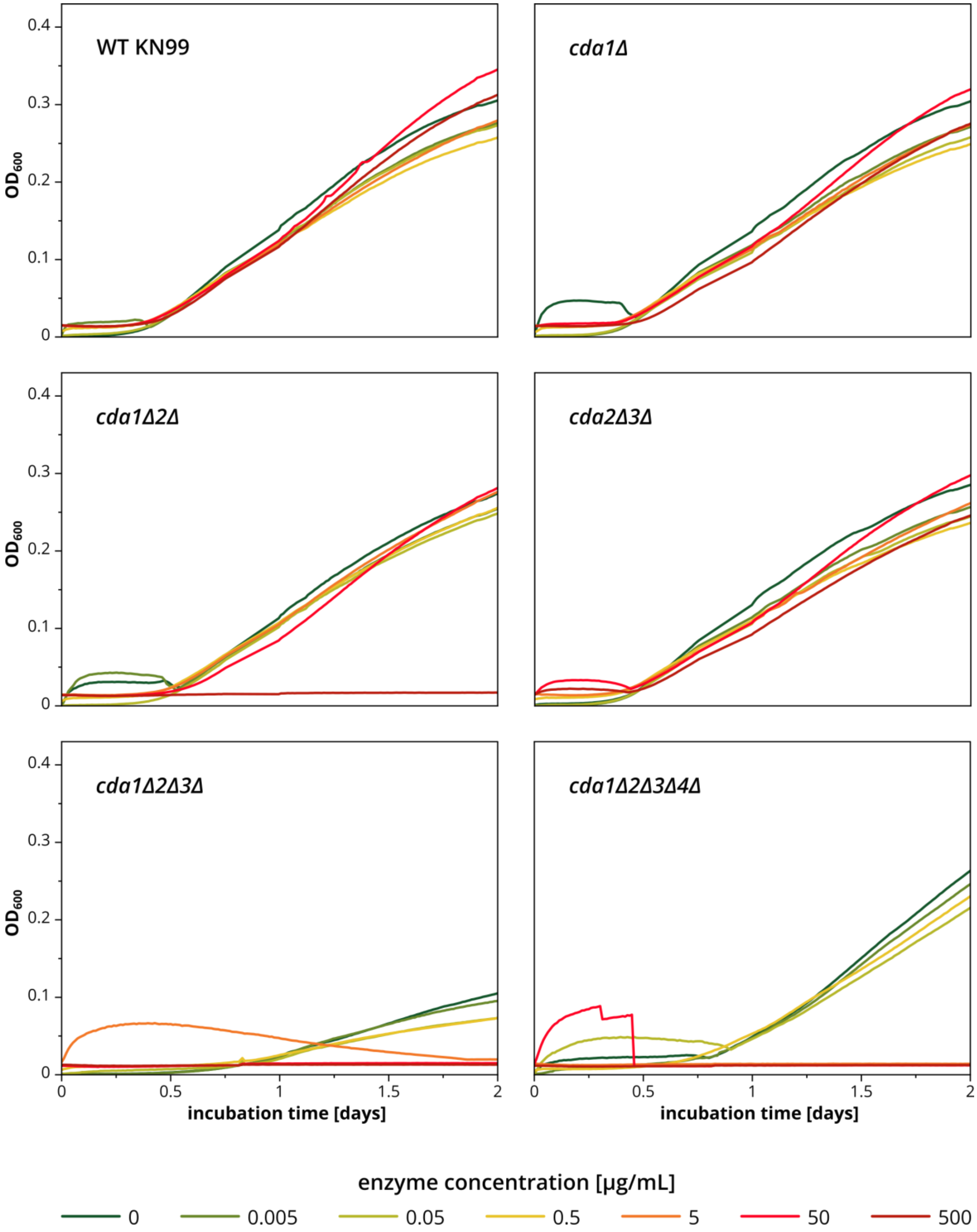

Fig. S7. Growth of selected strains over time in the presence of human lysozyme (HL). The indicated strains were cultivated in a 96-well plate in YNB-U medium at 30 °C in a spectrophotometer, and the OD_{600} was measured every 15 min. The different curves correspond to different concentrations of HL (expressed in rice) (Hellmann et al., 2025) and are means of N = 3 biological replicates.

Tables

Table S1. Data from previous publications describing the analysis of *Cryptococcus* chitosans compared with data from the present study. Blue font shows data from the indicated publications. Black font shows data from the present study. Red font indicates conditions (*e.g.*, temperature, duration, exact strain) of our data do not perfectly match those in the indicated publication. The following abbreviations are used for the publications: B05 (Banks et al., 2005), B07 (Baker et al., 2007), G12 (Gilbert et al., 2012), H20 (Hole et al., 2020), L19 (Lam et al., 2019), U18 (Upadhya et al., 2018), U21 (Upadhya et al., 2021), U23 (Upadhya et al., 2023). The conditional formatting applied to each column individually features a color scale from red (low) to yellow (intermediate) and green (high), allowing for the comparison of trends between our data and previous publications. This table is also available in xlsx format (allowing filtering, sorting, etc.) in the supporting information.

strain	medium	T [°C]	duration [h]	publication	nmole GlcNAc/ mg dry weight	nmole GlcNAc/ mg cell wall	nmole GlcN/ mg dry weight	nmole GlcN/ mg cell wall	FA	FA	mass fraction GlcNAc in dry weight [%(w/w)]	mass fraction GlcNAc in cell wall [%(w/w)]	mass fraction GlcN in dry weight [%(w/w)]	mass fraction GlcN in cell wall [%(w/w)]	mass fraction GlcNAc+GlcN in dry weight [%(w/w)]	mass fraction GlcNAc+GlcN in cell wall [%(w/w)]
WT H99	YPD	30	24	B05	10	n/a	60	n/a	0.14	n/a	0.2	n/a	1.0	n/a	1.2	n/a
WT H99	YPD	30	48	B05	25	150	90	90	0.22	0.62	0.5	3.1	1.4	1.5	2.0	4.5
WT KN99	YPD	30	24	L19	50	150	45	90	0.53	0.62	1.0	3.1	0.7	1.5	1.7	4.5
WT KN99	YPD	30	48	H20	n/a	150	150	90	n/a	0.62	n/a	3.1	2.4	1.5	n/a	4.5
WT KN99	YPD	30	48	L19	45	150	60	90	0.43	0.62	0.9	3.1	1.0	1.5	1.9	4.5
WT KN99	YPD	30	48	U18	n/a	150	125	90	n/a	0.62	n/a	3.1	2.0	1.5	n/a	4.5
WT KN99	YPD	30	48	U18	n/a	150	80	90	n/a	0.62	n/a	3.1	1.3	1.5	n/a	4.5
WT KN99	YPD	30	48	U21	n/a	150	120	90	n/a	0.62	n/a	3.1	1.9	1.5	n/a	4.5
WT KN99	YPD	30	48	U23	60	150	115	90	0.34	0.62	1.2	3.1	1.9	1.5	3.1	4.5
WT KN99	YPD	25	70	B07	25	150	65	90	0.28	0.62	0.5	3.1	1.0	1.5	1.6	4.5
WT KN99	YPD	30	72	L19	70	150	110	90	0.39	0.62	1.4	3.1	1.8	1.5	3.2	4.5
WT KN99	YPD	30	72	U18	n/a	150	80	90	n/a	0.62	n/a	3.1	1.3	1.5	n/a	4.5
WT KN99	YPD	30	96	L19	90	161	100	90	0.47	0.64	1.8	3.3	1.6	1.4	3.4	4.7
WT KN99	YPD	30	120	L19	50	161	120	90	0.29	0.64	1.0	3.3	1.9	1.4	2.9	4.7
WT KN99	YPD	30	?	G12	n/a	150	40	90	n/a	0.62	n/a	3.1	0.6	1.5	n/a	4.5
WT KN99	RPMI	37	120	L19	n/a	83	30	19	n/a	0.81	n/a	1.7	0.5	0.3	n/a	2.0
WT KN99	RPMI	37	120	U18	n/a	83	55	19	n/a	0.81	n/a	1.7	0.9	0.3	n/a	2.0
WT KN99	RPMI	37	120	U18	n/a	83	30	19	n/a	0.81	n/a	1.7	0.5	0.3	n/a	2.0
WT KN99	RPMI	37	120	U21	n/a	83	50	19	n/a	0.81	n/a	1.7	0.8	0.3	n/a	2.0
WT KN99	YNB-pH7	30	48	U23	75	37	60	25	0.56	0.59	1.5	0.8	1.0	0.4	2.5	1.2
WT KN99	YNB-U	30	48	U23	70	260	15	20	0.82	0.93	1.4	5.3	0.2	0.3	1.7	5.6
cda1Δ	YPD	30	48	U18	n/a	184	110	87	n/a	0.68	n/a	3.7	1.8	1.4	n/a	5.1
cda1Δ	YPD	30	48	U21	n/a	184	105	87	n/a	0.68	n/a	3.7	1.7	1.4	n/a	5.1
cda1Δ	YPD	30	72	U18	n/a	184	75	87	n/a	0.68	n/a	3.7	1.2	1.4	n/a	5.1
cda1Δ	RPMI	37	120	U18	n/a	142	30	17	n/a	0.90	n/a	2.9	0.5	0.3	n/a	3.1
cda1Δ	RPMI	37	120	U18	n/a	142	10	17	n/a	0.90	n/a	2.9	0.2	0.3	n/a	3.1
cda1Δ	RPMI	37	120	U21	n/a	142	30	17	n/a	0.90	n/a	2.9	0.5	0.3	n/a	3.1
cda2Δ	YPD	30	48	U18	n/a	207	115	95	n/a	0.68	n/a	4.2	1.9	1.5	n/a	5.7
cda2Δ	RPMI	37	120	U18	n/a	102	35	25	n/a	0.81	n/a	2.1	0.6	0.4	n/a	2.5
cda3Δ	YPD	30	48	U18	n/a	183	110	93	n/a	0.66	n/a	3.7	1.8	1.5	n/a	5.2

strain	medium	T [°C]	duration [h]	publication	nmole GlcNAc/ mg dry weight	nmole GlcNAc/ mg cell wall	nmole GlcN/ mg dry weight	nmole GlcN/ mg cell wall	FA	FA	mass fraction GlcNAc in dry weight [%(w/w)]	mass fraction GlcNAc in cell wall [%(w/w)]	mass fraction GlcN in dry weight [%(w/w)]	mass fraction GlcN in cell wall [%(w/w)]	mass fraction GlcNAc+GlcN in dry weight [%(w/w)]	mass fraction GlcNAc+GlcN in cell wall [%(w/w)]
cda3Δ	RPMI	37	120	U18	n/a	97	30	19	n/a	0.83	n/a	2.0	0.5	0.3	n/a	2.3
cda1Δ2Δ	YPD	30	48	U21	n/a	166	75	37	n/a	0.82	n/a	3.4	1.2	0.6	n/a	4.0
cda1Δ2Δ	YPD	25	70	B07	55	166	45	37	0.55	0.82	1.1	3.4	0.7	0.6	1.8	4.0
cda1Δ2Δ	RPMI	37	120	U21	n/a	158	10	8	n/a	0.95	n/a	3.2	0.2	0.1	n/a	3.3
cda1Δ3Δ	YPD	30	48	U21	n/a	128	90	49	n/a	0.72	n/a	2.6	1.4	0.8	n/a	3.4
cda1Δ3Δ	YPD	30	?	G12	n/a	128	30	49	n/a	0.72	n/a	2.6	0.5	0.8	n/a	3.4
cda1Δ3Δ	RPMI	37	120	U21	n/a	132	25	11	n/a	0.92	n/a	2.7	0.4	0.2	n/a	2.8
cda2Δ3Δ	YPD	30	48	U18	n/a	164	40	57	n/a	0.74	n/a	3.3	0.6	0.9	n/a	4.3
cda2Δ3Δ	YPD	30	48	U21	n/a	164	65	57	n/a	0.74	n/a	3.3	1.0	0.9	n/a	4.3
cda2Δ3Δ	RPMI	37	120	U21	n/a	100	30	17	n/a	0.85	n/a	2.0	0.5	0.3	n/a	2.3
cda1Δ2Δ3Δ	YPD	30	48	U18	n/a	168	0	5	n/a	0.97	n/a	3.4	0.0	0.1	n/a	3.5
cda1Δ2Δ3Δ	YPD	25	70	B07	120	168	0	5	1.00	0.97	2.4	3.4	0.0	0.1	2.4	3.5
cda1Δ2Δ3Δ	YPD	30	?	G12	n/a	168	0	5	n/a	0.97	n/a	3.4	0.0	0.1	n/a	3.5
cda1Δ2Δ4Δ	YPD	25	70	B07	60	138	55	33	0.52	0.81	1.2	2.8	0.9	0.5	2.1	3.3
cda1Δ2Δ3Δ4Δ	YPD	25	70	B07	125	205	0	6	1.00	0.97	2.5	4.2	0.0	0.1	2.5	4.3
chs3Δ	YPD	30	48	H20	n/a	118	5	5	n/a	0.96	n/a	2.4	0.1	0.1	n/a	2.5
chs3Δ	YPD	25	70	B07	70	118	5	5	0.93	0.96	1.4	2.4	0.1	0.1	1.5	2.5
WT *Cg* R265	YPD	30	24	L19	50	94	20	247	0.71	0.28	1.0	1.9	0.3	4.0	1.3	5.9
WT *Cg* R265	YPD	30	48	L19	45	94	90	247	0.33	0.28	0.9	1.9	1.4	4.0	2.4	5.9
WT *Cg* R265	YPD	30	72	L19	40	94	320	247	0.11	0.28	0.8	1.9	5.2	4.0	6.0	5.9
WT *Cg* R265	YPD	30	96	L19	60	103	280	252	0.18	0.29	1.2	2.1	4.5	4.1	5.7	6.2
WT *Cg* R265	YPD	30	120	L19	90	103	265	252	0.25	0.29	1.8	2.1	4.3	4.1	6.1	6.2
WT *Cg* R265	YPD	30	120	L19	n/a	94	225	247	n/a	0.28	n/a	1.9	3.6	4.0	n/a	5.9
WT *Cg* R265	YPD	30	120	L19	n/a	94	240	247	n/a	0.28	n/a	1.9	3.9	4.0	n/a	5.9
WT *Cg* R265	RPMI	37	120	L19	n/a	20	65	31	n/a	0.39	n/a	0.4	1.0	0.5	n/a	0.9
WT *Cg* R265	RPMI	37	120	L19	n/a	20	60	31	n/a	0.39	n/a	0.4	1.0	0.5	n/a	0.9
WT *Cg* R265	RPMI	37	120	L19	n/a	20	60	31	n/a	0.39	n/a	0.4	1.0	0.5	n/a	0.9
Cg cda1Δ	YPD	30	120	L19	n/a	n/a	150	n/a	n/a	n/a	n/a	n/a	2.4	n/a	n/a	n/a
Cg cda1Δ	RPMI	37	120	L19	n/a	n/a	40	n/a	n/a	n/a	n/a	n/a	0.6	n/a	n/a	n/a
Cg cda2Δ	YPD	30	120	L19	n/a	n/a	225	n/a	n/a	n/a	n/a	n/a	3.6	n/a	n/a	n/a
Cg cda2Δ	RPMI	37	120	L19	n/a	n/a	65	n/a	n/a	n/a	n/a	n/a	1.0	n/a	n/a	n/a
Cg cda3Δ	YPD	30	120	L19	n/a	n/a	220	n/a	n/a	n/a	n/a	n/a	3.5	n/a	n/a	n/a
Cg cda3Δ	RPMI	37	120	L19	n/a	n/a	10	n/a	n/a	n/a	n/a	n/a	0.2	n/a	n/a	n/a
Cg cda1Δ2Δ	YPD	30	120	L19	n/a	n/a	85	n/a	n/a	n/a	n/a	n/a	1.4	n/a	n/a	n/a
Cg cda1Δ2Δ	RPMI	37	120	L19	n/a	n/a	55	n/a	n/a	n/a	n/a	n/a	0.9	n/a	n/a	n/a
Cg cda1Δ3Δ	YPD	30	120	L19	n/a	n/a	105	n/a	n/a	n/a	n/a	n/a	1.7	n/a	n/a	n/a
Cg cda1Δ3Δ	RPMI	37	120	L19	n/a	n/a	5	n/a	n/a	n/a	n/a	n/a	0.1	n/a	n/a	n/a
Cg cda2Δ3Δ	YPD	30	120	L19	n/a	n/a	175	n/a	n/a	n/a	n/a	n/a	2.8	n/a	n/a	n/a
Cg cda2Δ3Δ	RPMI	37	120	L19	n/a	n/a	5	n/a	n/a	n/a	n/a	n/a	0.1	n/a	n/a	n/a
Cg cda1Δ2Δ3Δ	YPD	30	120	L19	n/a	n/a	5	n/a	n/a	n/a	n/a	n/a	0.1	n/a	n/a	n/a
Cg cda1Δ2Δ3Δ	RPMI	37	120	L19	n/a	n/a	5	n/a	n/a	n/a	n/a	n/a	0.1	n/a	n/a	n/a
WT *Sc*	YPD	30	?	B05	20	26	0	0	1.00	0.98	0.4	0.5	0.0	0.0	0.4	0.5

SI References

Baker, L.G., Specht, C.A., Donlin, M.J., Lodge, J.K., 2007. Chitosan, the deacetylated form of chitin, is necessary for cell wall integrity in Cryptococcus neoformans. Eukaryot. Cell 6, 855–867. https://doi.org/10.1128/EC.00399-06

Banks, I.R., Specht, C.A., Donlin, M.J., Gerik, K.J., Levitz, S.M., Lodge, J.K., 2005. A chitin synthase and its regulator protein are critical for chitosan production and growth of the fungal pathogen Cryptococcus neoformans. Eukaryot. Cell 4, 1902–1912. https://doi.org/10.1128/EC.4.11.1902-1912.2005

Bußwinkel, F., Goñi, O., Cord-Landwehr, S., O'Connell, S., Moerschbacher, B.M., 2018. Endochitinase 1 (Tv-ECH1) from *Trichoderma virens* has high subsite specificities for acetylated units when acting on chitosans. Int. J. Biol. Macromol. 114, 453–461. https://doi.org/10.1016/j.ijbiomac.2018.03.070

Gilbert, N.M., Baker, L.G., Specht, C.A., Lodge, J.K., 2012. A glycosylphosphatidylinositol anchor is required for membrane localization but dispensable for cell wall association of chitin deacetylase 2 in Cryptococcus neoformans. MBio 3, 1–8. https://doi.org/10.1128/mBio.00007-12

Hellmann, M.J., Marongiu, G.L., Gorzelanny, C., Moerschbacher, B.M., Cord-Landwehr, S., 2025. Hydrolysis of chitin and chitosans by the human chitinolytic enzymes: chitotriosidase, acidic mammalian chitinase, and lysozyme. Int. J. Biol. Macromol. 297, 139789. https://doi.org/10.1016/j.ijbiomac.2025.139789

Hole, C.R., Lam, W.C., Upadhya, R., Lodge, J.K., 2020. Cryptococcus neoformans Chitin Synthase 3 Plays a Critical Role in Dampening Host Inflammatory Responses. MBio 11, 1–13. https://doi.org/10.1128/mBio.03373-19

Lam, W.C., Upadhya, R., Specht, C.A., Ragsdale, A.E., Hole, C.R., Levitz, S.M., Lodge, J.K., 2019. Chitosan Biosynthesis and Virulence in the Human Fungal Pathogen *Cryptococcus gattii*. mSphere 4. https://doi.org/10.1128/mSphere.00644-19

Upadhya, R., Baker, L.G., Lam, W.C., Specht, C.A., Donlin, M.J., Lodge, J.K., 2018. *Cryptococcus neoformans* Cda1 and its chitin deacetylase activity are required for fungal pathogenesis. MBio 9, 1–19. https://doi.org/10.1128/mBio.02087-18

Upadhya, R., Lam, W.C., Hole, C.R., Parchment, D., Lee, C.K., Specht, C.A., Levitz, S.M., Lodge, J.K., 2021. Cryptococcus neoformans Cda1 and Cda2 coordinate deacetylation of chitin during infection to control fungal virulence. Cell Surf. 7, 100066. https://doi.org/10.1016/j.tcsw.2021.100066

Upadhya, R., Lam, W.C., Hole, C.R., Vasselli, J.G., Lodge, J.K., 2023. Cell wall composition in *Cryptococcus neoformans* is media dependent and alters host response, inducing protective immunity. Front. Fungal Biol. 4, 1–18. https://doi.org/10.3389/ffunb.2023.1183291
